# A Systems Biology Approach to Identify Essential Epigenetic Regulators for Specific Biological Processes in Plants

**DOI:** 10.3390/plants10020364

**Published:** 2021-02-13

**Authors:** Rachel M. McCoy, Russell Julian, Shoban R. V. Kumar, Rajeev Ranjan, Kranthi Varala, Ying Li

**Affiliations:** 1 Department of Horticulture and Landscape Architecture, Purdue University, West Lafayette, IN 47907, USA; mccoy26@purdue.edu (R.M.M.); russell.s.julian@gmail.com (R.J.); srajaman@purdue.edu (S.R.V.K.); ranjan9@purdue.edu (R.R.); kvarala@purdue.edu (K.V.); 2 Center for Plant Biology, Purdue University, West Lafayette, IN 47907, USA

**Keywords:** epigenetic regulator, machine learning, gene regulatory network, shoot apical meristem, root apical meristem

## Abstract

Upon sensing developmental or environmental cues, epigenetic regulators transform the chromatin landscape of a network of genes to modulate their expression and dictate adequate cellular and organismal responses. Knowledge of the specific biological processes and genomic loci controlled by each epigenetic regulator will greatly advance our understanding of epigenetic regulation in plants. To facilitate hypothesis generation and testing in this domain, we present EpiNet, an extensive gene regulatory network (GRN) featuring epigenetic regulators. EpiNet was enabled by (i) curated knowledge of epigenetic regulators involved in DNA methylation, histone modification, chromatin remodeling, and siRNA pathways; and (ii) a machine-learning network inference approach powered by a wealth of public transcriptome datasets. We applied GENIE3, a machine-learning network inference approach, to mine public Arabidopsis transcriptomes and construct tissue-specific GRNs with both epigenetic regulators and transcription factors as predictors. The resultant GRNs, named EpiNet, can now be intersected with individual transcriptomic studies on biological processes of interest to identify the most influential epigenetic regulators, as well as predicted gene targets of the epigenetic regulators. We demonstrate the validity of this approach using case studies of shoot and root apical meristem development.

## 1. Introduction

In eukaryotes, gene regulation occurs in the context of chromatin and nucleosomes, which is influenced by epigenetic modifications that pack genomic DNA and poise genes for activation or repression [1]. Broadly, epigenetic modifications include DNA methylation (which involves the siRNA silencing pathway), histone modification, and chromatin remodeling. Epigenetic states are dynamically modified by numerous epigenetic regulators that are encoded by specific gene families. For example, histone methylation, a form of histone modification, is added to specific amino acid residues on histone tails through the function of a group of enzymes called histone methyltransferases (HMTs) [2] and removed by the opposing action of another group of enzymes named histone demethylases (HDMs) [3]. Similarly, histone acetylation is maintained by the concerted actions of histone acetyltransferases (HAT) and histone deacetylases (HDAC) [3]. Epigenetic regulators can transform the chromatin landscape of a corresponding suite of genes to modulate their expression and dictate adequate cellular and organismal responses to developmental signals or environmental cues. Indeed, experimental evidence accumulated in the past 20 years established that epigenetic regulators and chromatin modifications play essential roles in modulating gene expression in response to developmental and environmental signals [3,4,5,6,7,8]. For example, SDG8, a H3K36 (lysine 36 of histone subunit 3) methyltransferase, was shown to specifically target hundreds of genes involved in defense, carbon metabolism, and reproductive development to modify their associated histones and gene expression levels [9], and it is required for proper responses to various environmental [10,11,12] and developmental signals [13]. To date, key epigenetic regulators have been identified for many, but not all, developmental and environmental processes [10,14,15,16,17,18,19]. Meanwhile, ongoing efforts to detect target genes of epigenetic regulators will continue to expand our knowledge of the specificity of epigenetic regulators [9,20]. A knowledge base to link each epigenetic regulator with specific biological processes and genome-wide targets is needed to move toward a genome-wide, systems-level understanding of epigenetic regulation.

Gene regulatory network (GRN) inference is a powerful method to predict regulatory relationships between regulators, genes, and biological processes. GRNs have been used to predict the regulatory relationships between transcription factors (TFs) and their target genes, and the TFs that control the most target genes in a GRN are identified as potential key regulators involved in the corresponding biological process [21,22]. Therefore, just as GRN inference has been used to infer transcriptional regulators [21,22], theoretically, it could be used to identify key epigenetic regulators of a given biological process—a notion which has not been tested, to the best of our knowledge. In eukaryotes, complex interplay between TFs and epigenetic regulators controls the coordinated expression of thousands of genes [23]. However, common approaches for gene network inference often use only TFs as regulators [24,25,26,27] and overlook the important layer of epigenetic regulation. In recent years, there has been an exponential accumulation of epigenomics data (e.g., in ENCODE [28]), which has led to emerging efforts to incorporate epigenetic states of genes (e.g., histone methylation status) into network models [29,30,31]. However, these models have not included the epigenetic regulators that cause these state changes in the resulting network models.

To facilitate the generation of knowledge on the specific function and targets of epigenetic regulators, we developed a bioinformatic pipeline built around GENIE3 [32] to mine large transcriptome datasets and construct tissue-specific GRNs with both epigenetic regulators and transcription factors as predictors or regulators of gene expression. Of the numerous GRN inference tools available [21], the random forest-based machine learning approach of GENIE3 was shown to be the best-performing tool to infer regulatory relationships from large transcriptome datasets [33]. The inferred GRNs can then be intersected with an individual transcriptomic study to identify the most influential epigenetic regulators for the biological processes of interest. Using meristematic development as a case study, we identified key epigenetic regulators that control a network of genes important for developmental processes in meristematic tissues of the shoot apical meristem (SAM) and root apical meristem (RAM), separately. Finally, our network predictions were broadly validated by published experimental data. The work presented here was based on *Arabidopsis thaliana* transcriptomic data, due to the abundance of this type of data in the public realm, but the described approaches could be applied to other plant systems. This bioinformatics pipeline can be applied to any biological process of interest to greatly speed up hypothesis generation and testing of the roles of epigenetic regulation in controlling plant developmental and environmental responses.

## 2. Results

### 2.1. Constructing Gene Regulatory Networks (GRNs) with Epigenetic Regulators

To probe the influence of epigenetic regulators in controlling GRNs, we first generated a list of known epigenetic regulators in model plant *Arabidopsis thaliana*. We chose to work with Arabidopsis because of the rich collection of knowledge on epigenetic regulators and a wealth of transcriptomic datasets is available for this model plant species. An extensive literature review was performed, and a list of 286 epigenetic regulators was manually curated based on 186 publications (see Table S1 for the complete list of genes and corresponding references). This includes 174 genes encoding epigenetic regulators involved in histone modification, 62 genes in DNA methylation, 30 in chromatin remodeling, and 20 involved in siRNA biogenesis (see Supplemental Results for description of these genes).

**Figure 1:**
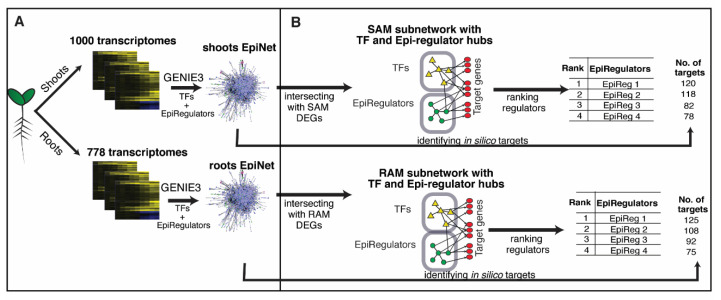
Workflow showing the analysis steps of this study: (**A**) 1000 transcriptomes from Arabidopsis shoots and 778 transcriptomes from Arabidopsis roots were downloaded from NCBI’s SRA (short read archive) database. GENIE3 was applied to construct gene regulatory networks from these transcriptomes for shoots and roots, separately, using both transcription factors (TFs) and epigenetic regulators (EpiRegulators) as predictors of gene expression levels. The resultant gene regulatory networks are named EpiNet, for shoots and roots, separately. (**B**) EpiNet was intersected with differentially expressed genes (DEGs) identified from individual transcriptomic studies to generate subnetworks relevant to the question of interest—for example, shoot apical meristem (SAM) and root apical meristem (RAM) development. The epigenetic regulators involved in controlling the subnetwork can then be ranked based on their influence on the genes in the subnetwork, to identify the most essential epigenetic regulators for the biological process of interest. For each epigenetic regulator, the *in silico* predicted targets can be identified in the EpiNet.

Next, we inferred organ-specific gene regulatory networks (GRNs) to determine regulatory relationships between a regulatory gene, i.e., an epigenetic regulator or transcription factor (TF), and its target genes. In detail, 1000 Arabidopsis transcriptomes from shoots and 778 transcriptomes from roots were retrieved from publicly available transcriptome data in SRA (short read archive, NCBI) (Figure 1A). Next, we ran GENIE3 [32], a machine learning algorithm that predicts gene regulatory networks from expression data [32], to infer the GRN for shoots and roots, separately (Figure 1A). Importantly, when running GENIE3, both transcription factors (1717 genes based on AtTFDB [34]) and epigenetic regulators (286 genes curated by this study, Table S1) were used as predictors, whose transcript abundances are used to predict the abundance of other transcripts (Figure 1A). Namely, both TFs and epigenetic regulators are allowed to influence the expression level of genes in the inferred network. The resultant GRNs contain nodes, which are genes including both predictors (TFs or epigenetic regulators) and regulated genes, and edges, where each edge represents a regulatory relationship between a predictor and a regulated gene. Each edge has a weight assigned by GENIE3, as a measurement of the confidence of the prediction [32]. All the edges in resultant shoot and root GRNs were ranked by weight (largest to smallest), separately, and the top 10% of edges were retrieved to generate higher confidence GRNs, referred to as EpiNet hereafter (Figure 1A). The shoot EpiNet includes 31,514 nodes and 6,286,230 edges, while the root EpiNet includes 33,134 nodes and 6,609,470 edges (Supplemental Data 1 and 2). In summary, by taking advantage of well-annotated epigenetic regulators and a wealth of transcriptome data in Arabidopsis, we have generated, with an unbiased approach, GRNs for shoots and roots. These GRNs were trimmed in an unbiased manner (retaining the top 10% of edges) to control their false positive rate [22,35,36]. Importantly, in these GRNs, in addition to TFs that are commonly used as predictors, epigenetic regulators are also included as predictors and thus contribute toward the regulation of a specific set of downstream target genes in the network.

### 2.2. Case Study: Identifying Influential Epigenetic Regulators in Meristematic Development

Next, we tested whether the EpiNet is able to provide informative predictions to understand the role of epigenetic regulators in specific biological processes. As a case study, we focused on the developmental processes of the shoot apical meristem (SAM) and root apical meristem (RAM). Plant meristems are the main area of primary growth and are made up of a collection of undifferentiated pluripotent stem cells, the number and positioning of which are under tight control. The SAM is the source of aboveground post-embryogenesis growth, including reproductive tissues, as it is converted to an inflorescence meristem during floral transition. The stem cells of the SAM are in the center of the meristem in an area termed the central zone (CZ). Surrounding these regions of slowly dividing cells is the peripheral zone (PZ) that gives rise to differentiated tissues. Below the CZ is the rib meristem (RM) zone that develops into stem tissue. Similarly, the RAM is the source of post-embryogenic root growth. The roots can be divided into the differentiated zone, the elongation zone, and the meristematic zone (which contains the RAM stem cells localized in the quiescent center (QC)). In general, meristem development has been well studied, and many transcription factors have been reported to perform important functions in maintaining meristematic tissues, including *WUSCHEL* and *KNOTTED1-LIKE HOMEOBOX* (*KNOX*) genes in SAM and *WUSCHEL-LIKE HOMEOBOX 5* (*WOX5*), *SCARECROW*, *SPATULA*, and *PLETHORA* in RAM (see [37,38,39,40] for recent reviews). By contrast, less is known about the roles of epigenetic regulators in SAM and RAM development [41,42]. Overall, SAM and RAM development provide a good model to test the efficacy of our approach.

#### 2.2.1. Creating Subnetworks Related to SAM and RAM Development Based on Representative Transcriptomic Studies

The EpiNet was generated without any focus on any particular biological processes and therefore contains transcriptional and epigenetic regulatory information for the whole genome. To generate subnetworks relevant to SAM or RAM development, we first searched for published transcriptomic studies that identified genes involved in SAM or RAM development. We focused on two transcriptomic studies of SAM, Yadav et al. [43] and Tian et al. [44]. In these two studies, cell-layer-specific transcriptomic profiling was performed; therefore, these studies likely have high sensitivity and identified a wide range of genes related to SAM development and function. In Yadav et al. [43], a mutant named *apetala1-1; cauliflower1-1* (*ap1-1; cal1-1*) was used because it produces multiple SAMs [45]. Different cell layers within the SAMs were labeled by expressing fluorescent proteins under the control of promoters known to drive gene expression specifically in those cell layers. For example, the *CLV3* promoter was used to label the central zone (CZ), the *FILAMENTOUSFLOWER* (*FIL*) promoter was used to label the peripheral zone (PZ), especially the flower organ primordia, and the *WUSCHEL* (*WUS*) promoter labeled the rib meristem (RM). Microarray assays were performed to compare the transcriptomes between the three labeled cell layers with fluorescent protein-negative SAM protoplasts as control. This study led to the identification of 2515 differentially expressed genes (DEGs) that are significantly upregulated in at least one of the three cell layers. Similarly, Tian et al. [44] also reported zone-specific transcriptomes from SAM through a different profiling method—translating ribosome affinity purification followed by sequencing (TRAP-Seq). In Tian et al. [44], transgenic plants were created in which the large subunit ribosomal protein L18 was fused with N-terminal His and FLAG tags, driven by zone-specific promoters such as p*CLV3*, p*UFO*, and p*WUS* (for CZ, PZ, and RM/organizing center (OC), respectively). From the transgenic seedlings, polysomes were immunoprecipitated using antibodies specific for the His or FLAG affinity tags, and the co-precipitated mRNA was then profiled by deep RNA-sequencing. This study identified 2013 genes that are expressed specifically in the CZ, PZ, and RM/OC zones in SAM.

For both studies, the list of identified DEGs was used to query the shoot EpiNet to generate a SAM subnetwork that contains: (i) epigenetic regulators and TFs that are inferred to affect the expression levels of the DEGs identified in Yadav et al. [43] or Tian et al. [44]; (ii) the DEGs that are regulated by the regulators in (i); and (iii) the regulatory relationships between (i) and (ii) (Figure 1B). Note that the regulators themselves are not required to be differentially expressed. From Yadav et al. [43], the resulting SAM subnetwork contains 482,082 edges and 4463 nodes, including 1673 TF regulators and 275 epigenetic regulators (Supplemental Data 3). From Tian et al. [44], the generated SAM subnetwork contains 358,047 edges and 3937 nodes, including 1650 TF regulators and 274 epigenetic regulators (Supplemental Data 4).

To generate the RAM subnetwork, we used RAM-specific DEGs reported in Nawy et al. [46]. In Nawy et al. [46], the *AGAMOUS-LIKE 42 (AGL42)* promoter, which is known to drive quiescent center (QC)-specific expression, was fused to GFP in order to label root cells in the QC. Fluorescent cells from root protoplasts were then sorted, and RNA was extracted and profiled using microarray. The global gene expression profile in the QC cells was compared to that in surrounding tissues, generating a list of 290 QC-specific expressed genes. This list of DEGs was used to query the root EpiNet, generating a RAM subnetwork with 67,533 edges and 2174 nodes, including 1612 TF regulators and 272 epigenetic regulators (Figure 1B; Supplemental Data 5).

#### 2.2.2. The Predicted Top TFs and Epigenetic Regulators Have Reported Roles in Meristem Development

To identify the most influential epigenetic regulators of SAM and RAM subnetworks, the regulators in each subnetwork were further ranked by their weight sum, which represents their net influence on the genes in the subnetwork (see Methods for details). The top ranked regulators with the greatest weight sums are predicted to regulate the largest number of genes in the subnetwork and/or have the highest confidence. We anticipate that some of the most influential regulators of apical meristem development, or indeed any well studied biological process, would have been already identified by traditional genetic approaches. Therefore, we first examined the top ranked regulators, including both TF regulators and epigenetic regulators, to determine whether known regulators of meristematic development are among the top predicted regulators in the SAM or RAM subnetworks. If ranked highly in the predicted list, such known regulators would support the efficacy of our network inference approach.

**Figure 2:**
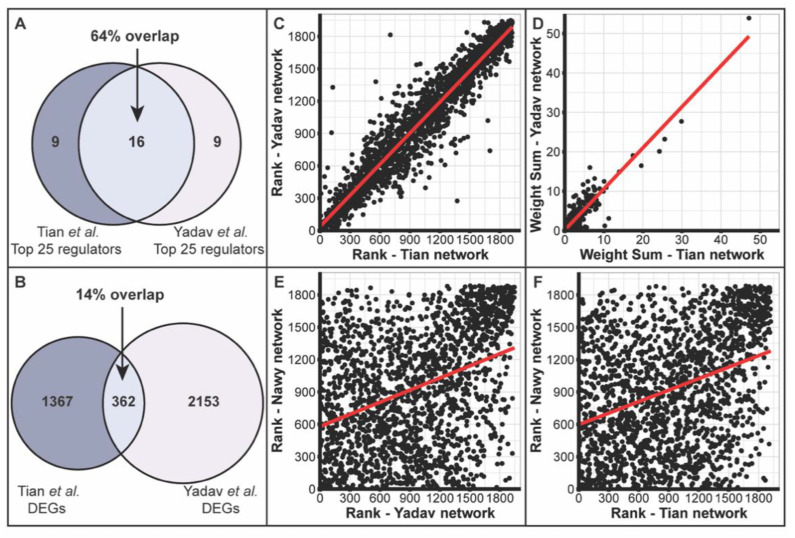
Similar regulators identified for SAM subnetworks generated from Yadav et al. and Tian et al. In total, 16 of the top 25 regulators are shared between the two SAM subnetworks (**A**), though they share only <20% of input DEG genes (**B**). The rank (**C**) and weight sum (**D**) of the two SAM subnetworks are strongly correlated (R^2^ = 0.9212 and 0.8875, respectively). By contrast, the correlation of regulator ranks between the RAM subnetwork (generated from Nawy et al.) with either the SAM subnetwork generated using Yadav et al. (**E**) or Tian et al. (**F**) is much weaker (R^2^ = 0.1292 and 0.1451, respectively).

We first examined the top 25 regulators identified in the two SAM subnetworks. Interestingly, the top 25 regulators identified based on Yadav et al. (Table S2) or Tian et al. (Table S3) share a 64% overlap (Figure 2A), even though the two lists of DEGs identified from Yadav et al. and Tian et al. only have a much smaller overlap (Figure 2B; an overlap of 362 genes, which represents 14.4% and 18.0% of the input DEG lists from Yadav et al. and Tian et al., respectively). Indeed, the regulators of the two SAM subnetworks share high similarity: the ranks (Figure 2C) and the weight sums (Figure 2D) of regulators are highly correlated between the two SAM subnetworks (R^2^ = 0.9212 for ranks and 0.8875 for weight sums). By contrast, the ranks of regulators are less correlated between the RAM subnetwork and the SAM subnetwork identified based on Yadav et al. (Figure 2E, R^2^ = 0.1451) or Tian et al. (Figure 2F, R^2^ = 0.1292).

To integrate the ranking of regulators from the analyses of Yadav et al. (Table S2) or Tian et al. (Table S3), a cumulative rank that summarizes the two lists of top 25 regulators was generated using a Rank Sum method (Table S4). In the integrated ranking, the top regulator is *HOMOLOGY DEPENDENT GENE SILENCING 1 (HOG1),* encoding a S-adenosyl-L-homocysteine hydrolase required for DNA methylation [47]. To the best of our knowledge, a role for HOG1 in meristematic development has not been reported. However, among the remaining top regulators, many regulators have been previously reported to be involved in regulating meristem development. For example, *CONSTANS-LIKE 5 (COL5)*, ranked second, is a member of the *CONSTANS* transcription factors family [48] and its overexpression was shown to induce flowering [49]. Our analysis thus suggests that COL5 possibly controls the SAM gene network to facilitate the transition of SAM to inflorescence meristem when environmental and/or developmental signals permit. In addition, two members of the *KNOTTED-LIKE HOMEOBOX (KNOX)* transcription factor family, *KNOTTED-LIKE FROM ARABIDOPSIS THALIANA 1 (KNAT1)* and *KNAT4*, are ranked fourth and seventh, respectively (Table S4). *KNAT1* is primarily expressed in and around the SAM [50], and overexpression of *KNAT1* results in lobed leaves with ectopic meristems [51]. *KNAT4* is reported to suppress meristematic capacity redundantly with other members of the KNOX family, *KNAT3* and *KNAT5* [52]. Further, *CYCLING DOF FACTOR 3 (CDF3)*, belonging to the DNA-binding with one finger (DOF)-type transcription factor family, is ranked sixth among the top regulators. CDF3 was shown to repress floral transition [53] and therefore might play a role in regulating the transition of SAM to inflorescent meristem. In addition to identifying previously known TFs involved in meristematic regulation, one epigenetic regulator with reported roles in regulating SAM development was also among the top regulator list (Table S4): *SPLAYED* (*SYD*; AT2G28290). SYD is a member of the SWI/SNF2 family of chromatin remodelers and has been shown to be required for meristem development, and *syd* mutants displayed premature termination of the floral meristem [42].

For RAM development, similarly, we examined the top ranked regulators, including both TF regulators and epigenetic regulators (Table S5), and identified multiple regulators with known functions in meristematic development. The top regulator in the RAM network is *WHIRLY 2 (WHY2)*, a member of the WHIRLY transcription factor family of single-stranded DNA binding proteins required for maintaining mitochondria. To the best of our knowledge, it is unknown whether WHY2 is involved in regulating RAM development. The second most influential regulator is *SPATULA* (*SPT*). *SPT* encodes a basic-helix-loop-helix family (bHLH) transcription factor first shown to be involved in floral development [54,55]. SPT was also reported as a negative regulator of organ size in leaves [56], cotyledons [57], and roots [58]. In roots, specifically, *spt* mutants have an increased number of cells in the QC and longer roots, suggesting that SPT is a repressor of cell division in RAM [58]. *STERILE APETALA (SAP),* ranked fourth, encodes an F-box protein that regulates proliferation of cells in the meristemoid, temporary stem cells which give rise to stomata [59,60]. The role of SAP in root meristems is therefore interesting and invites further investigation. In addition to the above TF regulators, a few epigenetic regulators with known functions in maintaining meristems have been also identified among the list of top 25 regulators (Table S5). *BRM* (AT2G46020), ranked 13th, encodes a SWI2/SNF2 chromatin remodeler [61] with a known role in RAM. *brm* mutants have smaller, disordered RAMs as BRM is required for proper maintenance of the QC [61]. Finally, two members of the plant-specific HD2 family of HDACs, HDT1 and HDT4, were among the top 25 regulators. Multiple members of the HD2 HDACs have been shown to regulate cell number in the root meristem [62].

Overall, the top TFs and epigenetic regulators identified in the SAM and RAM subnetworks are enriched with known regulators for meristematic development, including both TF regulators and epigenetic regulators. Our unbiased approach, with no prior knowledge of known regulators, thus assigned high ranks to many of the known regulators. These results provide robust support that our approach is capable of identifying the key regulators of any developmental processes. Therefore, the rest of the highly ranked but uncharacterized regulators are good candidates for novel regulators of SAM/RAM development.

#### 2.2.3. Identifying Highly Influential Epigenetic Regulators and Their Predicted Targets in SAM/RAM GRNs

Since our primary interest is to identify novel epigenetic regulators for specific biological processes, we generated the lists of top 10 epigenetic regulators in the SAM (Tables S6 and S7) and the RAM subnetworks (Table S8) (Figure 1B). For SAM, a cumulative rank that summarizes the two ranked lists based on either Yadav et al. (Table S6) or Tian et al. (Table S7) was generated by Rank Sum (Table S9). These lists provide promising candidates for novel epigenetic regulators of SAM/RAM development.

To further validate the specificity of our network predictions, the *in silico* network-inferred targets of top epigenetic regulators (referred to as “*in silico* targets” hereinafter) were retrieved from the EpiNet (Figure 1B; see Methods for details) and compared with experimentally detected targets of these epigenetic regulators, when available. Since the experimental data are often derived from whole plants and not constrained to the SAM and RAM tissues, we compared the experimentally defined targets to the genome-wide targets in EpiNet. The significance of the overlap is estimated using hypergeometric distribution (see Methods). Overall, we observed significant overlap between the *in silico* targets and experimentally validated targets for many top epigenetic regulators identified in our study (Table 1). For example, SYD is predicted to regulate 6797 genes in the shoot EpiNet. A transcriptomic study performed with the Arabidopsis ATH1 Affymetrix microarray identified 133 genes that are differentially expressed in the *syd-2* seedlings compared to wild-type (WT) plants [63]. Remarkably, 40 out of the 133 genes were predicted by our network, representing a significant enrichment of experimentally validated regulatory targets among the *in silico* targets (enrichment *p*- value < 0.048 determined by hypergeometric distribution against microarray background) (Table 1). Similarly, two genes encoding ubiquitin-conjugating enzymes (UBCs), *UBC1* and *UBC2*, were found among the top epigenetic regulators of SAM (Table S9). The UBCs are ubiquitin E2 conjugases that physically interact with two RING E3 ligases, HISTONE MONOUBIQUITATION 1 (HUB1) and HUB2, to monoubiquitinate H2B histones [64]. UBC1 and UBC2 were shown to regulate flowering time [64], possibly by affecting the transition from SAM to inflorescence meristem. In EpiNet, UBC1 and UBC2 are predicted to regulate 7239 and 6879 genes in the leaves, respectively. The *in silico* targets of UBC2 overlap significantly with genes that are downregulated in the *ubc1/ubc2* double mutants compared to WT in the context of salt-stress response [65] (32/82, hypergeometric *p* < 0.004, Table 1). Interestingly, the *in silico* targets of UBC1 do not overlap significantly with the experimentally determined misregulated genes in *ubc1/ubc2* (19/82, hypergeometric *p* = n.s., Table 1), possibly indicating that UBC2 has a more dominant role in salt stress than UBC1. Finally, *JUMONJI-C CONTAINING PROTEIN 30 (JMJ30)* has 4610 predicted *in silico* targets in the shoot network, which overlap significantly with genes with altered expression in *jmj30/jmj32* seedlings compared to WT plants [66] (32/74, hypergeometric *p* < 0.0005, Table 1).

In the roots, similarly, we observed significant overlaps between the *in silico* targets and experimentally determined targets for top epigenetic regulators. Interestingly, all four members of the plant-specific HD2 family of HDACs (*HDT1, HDT2, HDT3*, and *HDT4*) were among the top 10 epigenetic regulators of the RAM subnetwork (Table S8). The regulated genes by HDT1 and HDT2 in roots have been reported in a transcriptomic study comparing *hdt1,2* root tips with WT, specifically in a tissue-specific manner for the meristem zone, elongation zone, and differentiated zone separately [62]. Interestingly, the 8145 in silico targets of *HDT1* in the root EpiNet significantly overlap with genes differentially expressed in the meristem and elongation zones, but not the differentiated zone, in *hdt1,2* root tips (meristem 38/90, hypergeometric *p* < 0.007; elongation 47/114, hypergeometric *p* < 0.003; differentiated 17/42, hypergeometric *p* = n.s.; Table 1). There was no significant overlap between the experimentally determined regulated genes in *hdt1,2* root tips with the 8556 in silico targets of *HDT2* (Table 1). Another top epigenetic regulator is the chromatin remodeler *BRM*, which is predicted to regulate 5470 genes in the root EpiNet. These *in silico* targets significantly overlap with downregulated genes in seedlings of *brm-101* compared to WT [63] (26/93; hypergeometric *p* < 0.05 against microarray background, Table 1), as well as DEGs identified in leaves of *brm-1* mutants compared to WT [67] (925/4250, hypergeometric *p* < 8 × 10^−4^, Table 1). More importantly, the *in silico* targets of *BRM* also significantly overlap with direct targets of BRM identified *in planta* by ChIP-based genome-wide approaches in three studies: (i) two ChIP-Seq studies using transgenic lines carrying p*BRM::BRM-GFP* transgene in the *brm-1* mutant background (Li et al. [68] 1127/5278, hypergeometric *p* < 0.003, Table 1; Yu et al. [69] 1702/7761, hypergeometric *p* < 1.7 × 10^−7^, Table 1); and (ii) a ChIP-chip study using BRM antibody [70] (1032/4832, hypergeometric *p* < 0.004, Table 1). Finally, *PRMT4A* is predicted to regulate 5130 genes in the roots and these targets significantly overlap with DEGs identified in the *prmt4a/prmt4b* double mutant [71] (1082/5504, hypergeometric *p* < 0.01, Table 1).

Overall, we observed significant overlaps between the *in silico* network-inferred targets and experimentally validated targets, for the top epigenetic regulators identified in our study. These results further support the predictive power of the EpiNet approach. In summary, our study presented an unbiased gene regulatory network with epigenetic regulators incorporated as potential regulators, as well as a bioinformatics pipeline that allows for the identification and prioritization of influential epigenetic regulators for any specific biological process given that a DEG list is available for said biological process.

## 3. Discussion

Epigenetic regulators, working in concert with transcription factors to modify the expression levels of genes, have an emergent role in controlling gene networks underlying developmental and/or environmental responses. However, compared to transcription factors, the regulatory role of epigenetic modifiers is often overlooked in gene network inference. To integrate epigenomic data into networks, recent efforts have incorporated histone modification patterns as attributes of genes in a regulatory network [29,30]. Histone modifications have also been used as prior knowledge to improve predictive network models in yeast [31] However, these studies have overlooked the causal influence of epigenetic regulators on the expression levels of their genomic targets. Epigenetic regulators modify the expression level of their genomic targets, which has been shown in published studies in yeast and human [72,73] and in plants [9,74]. Specifically, epigenetic regulators could function as an “on” or “off” switch for a gene locus, e.g., via altering chromatin availability or modulating the abundance or quality of transcripts for actively transcribed genes. Epigenetic regulators could be recruited to these target gene loci by interacting with TFs [75] or other cis-motif-binding proteins [76]. Therefore, in our study, epigenetic regulators are allowed the same “predictor” status as TF regulators in gene network modeling. Including epigenetic regulators in gene network models will likely enhance the ability to predict network states under untested conditions.

The input data for our network inference were curated to only include gene expression values from either the shoots or the roots but in each case included a wide range of experimental conditions and stimuli. Therefore, the GRN inference is likely to pick up regulatory interactions that are broadly occurring in that organ and are not limited to a biological process of interest. No prior information was provided on the relative importance of regulators or the strength of known regulations to our approach, thus making it unbiased. As a result, it is able to predict known and unknown regulators of any biological process occurring in that organ. In our case study, the appropriate selection of a well-defined target set of genes, e.g., the genes involved in SAM/RAM maintenance and development, allows for the identification of TF and epigenetic regulators even when the underlying transcriptome data were not specifically chosen from meristematic tissues. Thus, the EpiNet approach was able to predict a GRN reasonably well in an untested, or at least underrepresented, condition of apical meristem maintenance.

In our study, we used DEGs generated from two different studies of SAM development, Yadav et al. [43] and Tian et al. [44], to create two SAM subnetworks. The two lists of DEGs only have a modest overlap, <20%, possibly reflecting the different experimental conditions (genotypes, biochemical methods for transcriptomic profiling, etc.) and/or informatics pipelines between the two studies (Figure 2B). Surprisingly, from the two reasonably different lists of DEGs, we identified highly similar regulators, evidenced by the 64% overlap of top 25 regulators (Figure 2A). Moreover, we observed a high correlation of ranks of regulators between the two subnetworks (Figure 2C). This cannot be explained merely by a dominant contribution from the overlapped 362 DEGs on the ranking of the regulators (Figure 2B), as in each case, the “unique” DEGs contribute to 85% and 82% of the total weight of edges of the subnetworks from Yadav et al. and Tian et al., respectively. Similarly, one might argue that a hub regulator in the whole EpiNet will likely remain a hub in any subnetwork, thus leading to the similar ranking of regulators between the two SAM subnetworks, but this does not appear to be the explanation here because the ranking of regulators in RAM is quite different (Figure 2E,F). Our observation likely suggests that although the identification of DEGs in a particular transcriptomic study could be sensitive to the specific experimental condition and biochemical/informatics protocols applied, the identification of the upstream epigenetic regulators, by contrast, is more robust.

Our analysis has generated insights into the epigenetic regulators underlying the complex developmental processes of the SAM and RAM. For example, all four members of the plant-specific HD2 family of HDACs (HDT1, 2, 3, and 4) were among the top 10 epigenetic regulators in the RAM subnetwork (Table S8). Similarly, in the SAM subnetwork, HDT1 and HDT2 are among the top epigenetic regulators (Table S9). This family of HDACs is unique in that it is plant-specific [77,78], and they are preferentially expressed in inflorescence tissues and relevant to environmental stresses [62,77,79,80]. HDT1 and HDT2 have been shown to regulate cell number in the root meristem [62]. Now, our analysis suggests that this family of HDACs is involved in regulating both SAM and RAM, possibly to alter the histone acetylation status of genes involved in meristematic development in response to environmental changes, potentially serving as an underlying mechanism of developmental plasticity to environmental changes in plants.

To validate our network prediction, we compared predicted *in silico* targets and experimentally detected targets, mostly determined through transcriptomic assays (Table 1). For multiple epigenetic regulators, we observed a significant overlap between the two types of target genes (*in silico* predicted vs. experimentally detected), suggesting that the computational predictions are comparable to the *in planta* scenarios. For a limited number of epigenetic regulators (e.g., BRM), their targets have also been identified by ChIP-based approaches. These ChIP-Seq and ChIP-chip studies have not been used in the construction of the EpiNet, which was built based on transcriptome data. Therefore, the significant overlaps between the *in silico* targets and the experimental targets identified by ChIP provide completely independent proof that the in silico predicted targets are likely enriched with *bona fide* targets. In addition, the overlaps of genes in some comparisons were likely underestimated, due to the limitation of experimental conditions. For example, transcriptomic profiling in Arabidopsis was often performed with whole seedlings; therefore, the transcriptomes are likely dominated by gene expression profiles from the shoots, with minimal representation from the roots. Therefore, the comparison of DEGs from such studies with the predicted targets in root EpiNet likely contains many false negatives. To reduce the false negative rate, future functional genomics studies should be performed in the roots to determine the specific gene targets of the epigenetic regulator in the roots. Indeed, the difference between the predicted targets and the experimentally validated targets could be caused by multiple reasons: (i) EpiNet predicts a full range of targets across multiple developmental stages and growth conditions, since the GRNs are based on hundreds to thousands of transcriptomic studies performed under various conditions. By contrast, the experimentally validated targets are limited to the specific developmental stages and growth conditions assayed in those particular experiments; (ii) the experimentally determined targets are sometimes limited by the platform used, e.g., microarray; (iii) the number of experimentally determined targets is also affected by the specific statistical cutoff used in the study; and (iv) additionally, GRN inference itself tolerates a modest to high false positive rate in order to improve recall (i.e., number of true positives identified) [22,33]. Nonetheless, our EpiNet approach demonstrates the strength of GRN-based, data-driven approaches in identifying functions of epigenetic regulators.

Overall, we expect that the EpiNet database and pipeline presented in this study could be utilized by the plant community in a couple of ways: (i) the EpiNet can be intersected with DEGs generated through any transcriptomic study relevant to any specific biological process of interest, to identify the epigenetic regulators that are most important for this process, in order to speed up hypothesis generation and testing; (ii) since experimental determination of genome-wide targets of epigenetic regulators is time- and resource-consuming, as a preliminary study, any epigenetic regulator of interest could be queried in the EpiNet databases to identify the predicted targets for shoots and roots, separately. We used SAM and RAM development as examples, and the described approaches can be expanded to study other processes. Our EpiNet was built with transcriptome data from Arabidopsis to take advantage of the extensive collection of transcriptomes generated for this model plant, as training GRNs generally benefits from a large input dataset of transcriptomes. For other plant systems with >1000 transcriptomes available, the approaches reported in this study could be applied to generate a species-specific EpiNet. Otherwise, the Arabidopsis EpiNet could still be queried through ortholog mapping. Overall, knowledge of how different epigenetic regulators regulate specific developmental or environmental processes will greatly expand our understanding of gene regulation in plants.

## 4. Materials and Methods

### 4.1. Construction of Tissue-Specific Gene Regulatory Networks Including Epigenetic Regulators

The shoot and root RNA-Seq datasets from *Arabidopsis thaliana* were obtained from NCBI SRA. The root set included 778 individual RNA-Seq runs and 1000 individual RNA-Seq runs were retained for the shoot. In both cases, the expression level of all genes across all runs was used to infer a gene regulatory network using the R implementation of the GENIE3 algorithm [32] with the parameters K = all and nCores = 24 and using R version 3.5.1. The computation was performed on a Linux server with 24 cores (Sky lake CPUs @ 2.60Hz), 384 GB RAM. Each inference job took multiple (~7–10) days to complete. The set of regulators included the known 1717 TFs in Arabidopsis (AtTFDB [34]) as well as 286 epigenetic regulators described above. In the generated network, the weight of the edge is a measure of the confidence in that regulatory edge relative to all the inferred edges in the network [32]. From the ranked list of predictor->target weighted edges provided by GENIE3, edges with the top 10% of weights were retained for further analysis, referred to as EpiNet. The top 10% of predicted network is an arbitrary threshold chosen to reduce the low confidence and, often, false positive edges common to network prediction.

### 4.2. Creating Subnetworks of SAM and RAM Development and Identifying the Top Regulators

Genes specifically expressed in the RAM (Nawy et al. [46]) or SAM (Yadav et al. [43] and Tian et al. [44]) were used to filter the EpiNet generated by GENIE3. Each edge of EpiNet (i.e., each line of the GENIE3 output) includes a Regulatory Gene (i.e., regulator or predictor), a Target Gene, and a Weight. To generate SAM- or RAM-specific subnetworks, the shoot or root EpiNet was filtered to only include edges where the Target Gene was in the experimentally determined DEG list. Next, an in-house python script was run to sum the weight of all edges in the subnetwork for every regulator to generate a weight sum. Regulatory genes were then ranked by weight sum from highest to lowest. To determine the top 10 predicted epigenetic regulators, the list of epigenetic regulators (Table S1) was used to filter the rank of Regulatory Genes.

### 4.3. Assessing the Significance of the Overlap between In Silico Predicted Targets and Experimentally Validated Targets of Influential Epigenetic Regulators

To determine the *in silico* targets of an epigenetic regulator, an in-house python script was used to select all edges in the shoot or root EpiNet, where the Regulatory Gene is the epigenetic regulator of interest. The collection of the Target Genes from these selected edges then constitutes the *in silico* targets of the epigenetic regulator. The significance of overlap between *in silico* and experimental targets was determined using hypergeometric distribution in R statistical programming software (phyper; lower.tail = FALSE for overrepresentation). Unless otherwise mentioned, the total number of genes in Arabidopsis was used as background. When experimental data were from a microarray experiment, *in silico* targets were filtered to only include genes presented on the microarray, and the number of probes was used as background.

## 5. Conclusions

We present EpiNet, a bioinformatics resource for inferring organ-specific regulatory relationships between epigenetic regulators and their targets. EpiNet could be used to identify important epigenetic regulators of a specific biological process, as well as regulatory targets of these epigenetic regulators, thus facilitating generation of hypotheses on epigenetic regulation. EpiNet utilizes the well-annotated genome and wealth of available transcriptome studies from Arabidopsis, but this approach could be used in other plants with sufficient transcriptome data.

## Figures and Tables

**Table 1:** Validation of *in silico* target predictions of epigenetic regulators in shoots and roots. For each epigenetic regulator (1st column), the *in silico* targets from EpiNet (2nd column) were compared with experimentally determined targets (3rd column) to determine the size of overlap (4th column). Hypergeometric testing was used to determine whether the overlap was statistically significant compared to a random background, and the *p*-value is reported (5th column). A brief description of the experimental data and the citation is included in the last column.

	Epigenetic Regulator	No. of In Silico Targets	No. of Experimental Targets	No. of Overlapping Targets	Overlap *p*-Value	Sources of Experimental Data
**Shoots**	SYD	4936 ^1^	133	40	0.0476	DEGs in *syd-2* seedlings [63]
	UBC1	7239	82	19	0.7693	Salt stress-induced genes with lower expression in *ubc1*/*ubc2* [65]
	UBC2	6879	82	32	0.0032	Salt stress-induced genes with lower expression in *ubc1*/*ubc2* [65]
	JMJ30	4642	106	32	0.0004	DEGs in *jmj30*/*jmj32* seedlings [66]
**Roots**	HDT1	8145	90	38	0.0066	DEGs in meristem zone of *hdt1*/*2* root tips [62]
			114	48	0.0027	DEGs in elongation zone of *hdt1*/*2* root tips [62]
			42	8	0.9557	DEGs in differentiated zone of *hdt1*/*2* root tips [62]
	HDT2	8556	90	34	0.0996	DEGs in meristem zone of *hdt1*/*2* root tips [62]
			114	42	0.1041	DEGs in elongation zone of *hdt1*/*2* root tips [62]
			42	17	0.1220	DEGs in differentiated zone of *hdt1*/*2* root tips [62]
	BRM	4234 ^1^	93	26	0.0436	Downregulated genes in *brm-101* seedlings [63]
	BRM	5470	4250	925	0.0003	DEGs in *brm-1* leaves [67]
			5278	1127	0.0008	ChIP-Seq using *pBRM::BRM-GFP* in *brm-1* [68]
			7761	1702	<0.0001	ChIP-Seq using *pBRM::BRM-GFP* in *brm-1* [69]
			4831	1032	0.0014	ChIP-chip study using BRM antibody [70]
	PRMT4A	5130	5504	1082	0.0098	DEGs in *prmt4a*/*prmt4b* seedlings [71]

^1^: *in silico* targets were filtered based on microarray probes.

## Data Availability

Data supporting reported results can be found in supplemental materials listed above.

## Supplementary Materials

The following are available online at https://www.mdpi.com/2223-7747/10/2/364/s1: Supplemental results: Description and literature review of epigenetic regulators used as predictors; Supplementary data S1: Shoot EpiNet; Supplementary data S2: Root EpiNet; Supplementary data S3: SAM subnetwork based on DEGs from Yadav et al.; Supplementary data S4: SAM subnetwork based on DEGs from Tian et al.; Supplementary data S5: RAM subnetwork based on DEGs from Nawy et al.; Supplementary Table S1: List of epigenetic regulators used in this study and references; Supplementary Table S2: Top 25 regulators in the SAM subnetwork generated from Yadav et al. 2009; Supplementary Table S3: Top 25 regulators in the SAM subnetwork generated from Tian et al. 2019; Supplementary Table S4: Cumulative ranking of the top regulators from the SAM subnetworks generated from Yadev et al. and Tian et al.; Supplementary Table S5: Top 25 regulators in the RAM subnetwork generated from Nawy et al. 2009; Supplementary Table S6: Top 10 epigenetic regulators in the SAM subnetwork generated from Yadav et al. 2009; Supplementary Table S7: Top 10 epigenetic regulators in the SAM subnetwork generated from Tian et al. 2019; Supplementary Table S8: Top 10 epigenetic regulators in the RAM subnetwork generated from Nawy et al. 2008; Supplementary Table S9: Cumulative ranking of the top epigenetic regulators in the SAM subnetworks generated from Yadav et al. and Tian et al.
