# Supplementary material for: A Systems Biology Approach to Identify Essential Epigenetic Regulators for Specific Biological Processes in Plants
Source: Plants (Basel). 2021 Feb 13;10(2):364. doi: 10.3390/plants10020364 (PMC7918732; doi:10.3390/plants10020364)
Supplement: Supplementary file 1 [file plants-10-00364-s001.zip › Supplemental Table S1_Final.docx]

**Table 1. List of epigenetic regulators used for network inference in this study.**

| **Gene ID** | **Gene Name** | **Class** | **Reference** |
| --- | --- | --- | --- |
| AT2G31650 | ATX1 | histone modification | [1] |
| AT5G58230 | MSI1 | histone modification | [2] |
| AT1G21710 | OGG1 | DNA methylation | [3] |
| AT2G22740 | SUVH6 | histone modification | [4] |
| AT3G10010 | DML2 | DNA methylation | [5] |
| AT4G34060 | DML3 | DNA methylation | [5] |
| AT5G04290 | KTF1 | DNA methylation | [6] |
| AT4G19020 | CMT2 | DNA methylation | [7] |
| AT5G66750 | CHR1/DDM1 | chromatin remodeling | [7] |
| AT2G24490 | RPA2 | histone modification | [8] |
| AT3G23780 | NRPE2 | DNA methylation | [9] |
| AT5G13960 | SUVH4/KYP | histone modification | [10] |
| AT4G00416 | MBD3 | DNA methylation | [11] |
| AT5G50320 | ELO3/HAG3 | histone modification | [12] |
| AT5G17690 | LHP1 | histone modification | [2] |
| AT2G27040 | AGO4 | DNA methylation/ siRNA biogenesis | [13] |
| AT5G21150 | AGO9 | siRNA biogenesis | [14] |
| AT4G38130 | HDA19 | DNA methylation | [15] |
| AT2G19670 | PRMT1A | histone modification | [16] |
| AT4G29510 | PRMT1B | histone modification | [16] |
| AT5G23570 | SGS3 | siRNA biogenesis | [17] |
| AT3G12270 | PRMT3 | histone modification | [18] |
| AT1G50410 | FRG2 | chromatin remodeling/ DNA methylation | [19] |
| AT3G20010 | FRG1 | chromatin remodeling /DNA methylation | [19] |
| AT3G14890 | ZDP | DNA methylation | [20] |
| AT3G19040 | HAF2 | histone modification | [21] |
| AT5G20930 | TOUSLED | siRNA biogenesis | [22] |
| AT3G49500 | RDR6 | siRNA biogenesis | [23] |
| AT2G35160 | SUVH5 | histone modification | [24] |
| AT4G11130 | RDR2 | DNA methylation/ siRNA biogenesis | [25] |
| AT2G47620 | SWI3A | chromatin remodeling | [26] |
| AT2G28290 | SYD | chromatin remodeling | [27] |
| AT3G42670 | CLSY/CHR38 | chromatin remodeling/ DNA methylation | [28] |
| AT3G44490 | HDA17 | histone modification | [29] |
| AT1G18450 | ARP4 | chromatin remodeling | [30] |
| AT3G06010 | CHR12 | chromatin remodeling | [31] |
| AT2G13370 | CHR5 | chromatin remodeling | [32] |
| AT4G31120 | PRMT5/SKB1 | histone modification | [33] |
| AT5G18620 | CHR17 | chromatin remodeling | [34] |
| AT3G03300 | DCL2 | siRNA biogenesis | [35] |
| AT1G69770 | CMT3 | DNA methylation | [36] |
| AT3G10390 | FLD | histone modification | [37] |
| AT1G63020 | NRPD1A | DNA methylation | [38] |
| AT2G40030 | NRPE1 | DNA methylation | [38] |
| AT2G43410 | FPA | siRNA biogenesis | [39] |
| AT4G16280 | FCA | siRNA biogenesis | [39] |
| AT3G07610 | IBM1/JMJ25 | histone modification | [40] |
| AT3G14980 | ROS4/IDM1 | histone modification | [41] |
| AT1G19100 | MORC6 | DNA methylation/ chromatin remodeling | [42] |
| AT4G36290 | MORC1 | DNA methylation/ chromatin remodeling | [42] |
| AT1G02580 | MEA | histone modification | [43] |
| AT3G21820 | ATXR2 | histone modification | [44] |
| AT1G77300 | ASHH2/SDG8 | histone modification | [45] |
| AT5G19310 | CHR23 | chromatin remodeling | [46] |
| AT3G06930 | PRMT4B | histone modification | [47] |
| AT5G49020 | PRMT4A | histone modification | [47] |
| AT5G43810 | AGO10 | siRNA biogenesis | [48] |
| AT3G12810 | PIE1 | chromatin remodeling | [49] |
| AT3G33520 | ARP6 | chromatin remodeling | [49] |
| AT5G37055 | SWC6 | chromatin remodeling | [49] |
| AT2G46020 | BRM | chromatin remodeling | [50] |
| AT1G65470 | FAS1 | chromatin remodeling/ assembly | [51] |
| AT5G64630 | FAS2 | chromatin remodeling | [51] |
| AT1G09700 | HYL1 | siRNA biogenesis | [52] |
| AT1G57820 | VIM1 | DNA methylation | [53] |
| AT1G66050 | VIM2 | DNA methylation | [53] |
| AT5G39550 | VIM3 | DNA methylation | [53] |
| AT3G17310 | DRM3 | DNA methylation | [54] |
| AT3G20740 | FIE | histone modification | [55] |
| AT2G30280 | RDM4 | DNA methylation | [56] |
| AT2G19520 | FVE | histone modification | [57] |
| AT5G51230 | EMF2 | histone modification | [58] |
| AT5G48120 | MET18 | DNA methylation | [59] |
| AT1G31280 | AGO2 | siRNA biogenesis | [60] |
| AT2G32940 | AGO6 | DNA methylation/ siRNA biogenesis | [61] |
| AT1G01040 | DCL1 | siRNA biogenesis/ DNA methylation | [62] |
| AT3G60830 | ARP7 | chromatin remodeling | [63] |
| AT1G55970 | HAC4 | histone modification | [64] |
| AT1G04870 | PRMT10 | histone modification | [65] |
| AT1G73150 | GTE3 | histone modification | [66] |
| AT5G63110 | HDA6 | histone modification | [67] |
| AT2G34880 | MEE27 | histone modification | [68] |
| AT3G11200 | ALF2 | histone modification | [69] |
| AT5G20510 | ALF5 | histone modification | [69] |
| AT1G17790 | GTE5 | histone modification | [70] |
| AT3G01770 | BET10/GTE11 | histone modification | [70] |
| AT3G27260 | GTE8 | histone modification | [70] |
| AT3G52280 | GTE6 | histone modification | [70] |
| AT5G10550 | GTE2 | histone modification | [70] |
| AT5G14270 | BET9/GTE9 | histone modification | [70] |
| AT5G46550 | GTE12 | histone modification | [70] |
| AT5G65630 | GTE7 | histone modification | [70] |
| AT1G09450 | Haspin-like gene | histone modification | [71] |
| AT3G04810 | NIMA kinase | histone modification | [71] |
| AT3G12200 | NIMA kinase | histone modification | [71] |
| AT3G20860 | NIMA kinase | histone modification | [71] |
| AT3G44200 | NIMA kinase | histone modification | [71] |
| AT3G63280 | NIMA kinase | histone modification | [71] |
| AT5G28290 | NIMA kinase | histone modification | [71] |
| AT1G26760 | ATXR1 | histone modification | [72] |
| AT1G11950 | JMJ26 | histone modification | [73] |
| AT1G62310 | JMJ29 | histone modification | [73] |
| AT1G63490 | JMJ17 | histone modification | [73] |
| AT1G78280 | JMJ21 | histone modification | [73] |
| AT2G38950 | JMJ19 | histone modification | [73] |
| AT3G45880 | JMJ32 | histone modification | [73] |
| AT4G21430 | JMJ28 | histone modification | [73] |
| AT5G06550 | JMJ22 | histone modification | [73] |
| AT5G19840 | JMJ31 | histone modification | [73] |
| AT5G63080 | JMJ20 | histone modification | [73] |
| AT5G25480 | DNMT2 | DNA methylation | [74] |
| AT2G16390 | DRD1/DMS1 | chromatin remodeling/ DNA methylation | [75] |
| AT1G06770 | AtRING1B | histone modification | [76] |
| AT2G30580 | DRP2/BMI1A | histone modification | [76] |
| AT3G17590 | BSH | chromatin remodeling | [77] |
| AT3G57300 | INO80 | chromatin remodeling | [78] |
| AT1G05460 | SDE3 | siRNA biogenesis | [79] |
| AT1G54840 | IDM2 | DNA methylation | [80] |
| AT1G20870 | IDM3 | DNA methylation | [81] |
| AT5G21030 | AGO8 | siRNA biogenesis | [82] |
| AT3G20020 | PRMT6 | histone modification | [83] |
| AT4G16570 | PRMT7 | histone modification | [83] |
| AT1G15340 | MBD10 | DNA methylation | [11] |
| AT1G22310 | MBD8 | DNA methylation | [11] |
| AT3G15790 | MBD11 | DNA methylation | [11] |
| AT3G46580 | MBD5 | DNA methylation | [11] |
| AT3G63030 | MBD4 | DNA methylation | [11] |
| AT4G22745 | MBD1 | DNA methylation | [11] |
| AT5G35330 | MBD02 | DNA methylation | [11] |
| AT5G35338 | MBD12 | DNA methylation | [11] |
| AT5G52230 | MBD13 | DNA methylation | [11] |
| AT5G59380 | MBD6 | DNA methylation | [11] |
| AT4G30860 | SDG4 | histone modification | [84] |
| AT4G16845 | VRN2 | histone modification | [85] |
| AT5G04560 | DME | DNA methylation | [86] |
| AT2G36490 | DML1 | DNA methylation | [87] |
| AT5G49160 | MET1 | DNA methylation | [88] |
| AT5G14620 | DRM2 | DNA methylation | [89] |
| AT5G15380 | DRM1 | DNA methylation | [89] |
| AT4G20910 | HEN1 | DNA methylation/ siRNA biogenesis | [90] |
| AT4G14140 | DMT2 | DNA methylation | [91] |
| AT2G23380 | CLF | histone modification | [92] |
| AT4G20400 | JMJ14 | histone modification | [93] |
| AT1G04050 | SUVR1 | histone modification | [94] |
| AT5G43990 | SUVR2 | histone modification | [94] |
| AT1G72270 | HDP1 | DNA methylation | [95] |
| AT4G31270 | HDP2 | DNA methylation | [95] |
| AT1G69440 | AGO7 | siRNA biogenesis | [96] |
| AT5G58130 | ROS3 | DNA methylation | [97] |
| AT3G22680 | RDM1 | DNA methylation | [98] |
| AT5G04240 | ELF6 | histone modification | [99] |
| AT1G05910 | BRAT1 | histone modification | [100] |
| AT3G48430 | REF6 | histone modification | [101] |
| AT5G06600 | UBP12 | histone modification | [102] |
| AT1G31290 | AGO3 | siRNA biogenesis | [103] |
| AT5G09790 | ATXR5 | histone modification | [104] |
| AT5G24330 | ATXR6 | histone modification | [104] |
| AT3G48670 | IDN2 | DNA methylation | [105] |
| AT3G49250 | DMS3/IDN1 | DNA methylation | [105] |
| AT1G09320 | ADCP1 | histone modification | [106] |
| AT5G46910 | JMJ13 | histone modification | [107] |
| AT1G48410 | AGO1 | siRNA biogenesis | [108] |
| AT3G06400 | CHR11 | chromatin remodeling | [109] |
| AT4G12620 | ORC1B | histone modification | [110] |
| AT4G14700 | ORC1A | histone modification | [110] |
| AT4G15180 | SDG2/ATXR3 | histone modification | [111] |
| AT2G27350 | OTLD1 | histone modification | [112] |
| AT3G20810 | JMJ30 | histone modification | [113] |
| AT5G11470 | IBM2/ASI1 | DNA methylation | [114] |
| AT5G55390 | EDM2 | histone modification | [115] |
| AT2G44150 | ASHH3 | histone modification | [116] |
| AT3G52250 | PWR | histone modification | [117] |
| AT5G03740 | HD2C | histone modification | [118] |
| AT2G25170 | PKL | chromatin remodeling | [119] |
| AT4G31900 | CHR7 | chromatin remodeling | [119] |
| AT5G44800 | CHR4 | chromatin remodeling | [119] |
| AT3G44680 | HDA9 | histone modification | [120] |
| AT2G33610 | SWI3B | chromatin remodeling | [121] |
| AT1G58025 | BRD5 | histone modification | [122] |
| AT1G61215 | BRD4 | histone modification | [122] |
| AT1G76380 | BRD2 | histone modification | [122] |
| AT2G44430 | BRD3 | histone modification | [122] |
| AT3G60110 | BRD6 | histone modification | [122] |
| AT5G49430 | BRD11 | histone modification | [122] |
| AT5G55040 | BRD13 | histone modification | [122] |
| AT1G13790 | FDM4 | DNA methylation | [123] |
| AT1G15910 | FDM1 | DNA methylation | [123] |
| AT1G80790 | FDM5 | DNA methylation | [123] |
| AT3G12550 | FDM3 | DNA methylation | [123] |
| AT4G00380 | FDM2 | DNA methylation | [123] |
| AT3G43920 | DCL3 | DNA methylation/ siRNA biogenesis | [124] |
| AT5G20320 | DCL4 | siRNA biogenesis | [124] |
| AT1G02740 | MRG2 | histone modification | [125] |
| AT4G37280 | MRG1 | histone modification | [125] |
| AT5G04940 | SUVH1 | histone modification | [126] |
| AT2G27840 | HDT4 | histone modification | [127] |
| AT3G18520 | HDA15 | histone modification | [128] |
| AT1G79000 | HAC1 | histone modification | [129] |
| AT3G12980 | HAC5 | histone modification | [129] |
| AT5G63320 | NPX1 | histone modification | [130] |
| AT3G01460 | MBD9 | histone modification | [131] |
| AT1G17770 | SUVH7 | histone modification | [132] |
| AT1G76710 | ASHH1 | histone modification | [132] |
| AT2G19640 | ASHR2 | histone modification | [132] |
| AT3G03750 | SUVR3 | histone modification | [132] |
| AT3G59960 | ASHH4 | histone modification | [132] |
| AT4G02020 | SWN | histone modification | [132] |
| AT3G49600 | UBP26 | histone modification | [133] |
| AT1G06230 | GTE4 | histone modification | [134] |
| AT5G35600 | HDA7 | histone modification | [135] |
| AT1G21700 | SWI3C | chromatin remodeling | [136] |
| AT5G59800 | MBD7 | DNA methylation | [137] |
| AT1G54390 | ING2 | histone modification | [138] |
| AT5G11530 | EMF1 | histone modification | [139] |
| AT5G57380 | VIN3 | histone modification | [140] |
| AT3G61740 | SDG14/ATX3 | histone modification | [141] |
| AT4G27910 | SDG16/ATX4 | histone modification | [141] |
| AT5G53430 | SDG29/ATX5 | histone modification | [141] |
| AT1G32810 | AT1G32810 | histone modification | [142] |
| AT4G13940 | HOG1 | DNA methylation | [143] |
| AT2G25880 | aurora H3 kinase | histone modification | [144] |
| AT2G45490 | aurora H3 kinase | histone modification | [144] |
| AT4G32830 | aurora H3 kinase | histone modification | [144] |
| AT4G34430 | SWI3D | chromatin remodeling | [26] |
| AT5G26040 | HDA2 | histone modification | [145] |
| AT1G55250 | HUB2 | histone modification | [146] |
| AT2G44950 | HUB1 | histone modification | [146] |
| AT1G62830 | LDL1/KDM1C | histone modification | [147] |
| AT3G13682 | LDL2 | histone modification | [147] |
| AT4G16310 | LDL3 | histone modification | [147] |
| AT3G44600 | CYP71 | histone modification | [148] |
| AT1G05830 | ATX2 | histone modification | [149] |
| AT5G42400 | ATXR7 | histone modification | [150] |
| AT5G61070 | HDA18 | histone modification | [151] |
| AT3G01320 | SNL1 | histone modification | [152] |
| AT5G15020 | SNL2 | histone modification | [152] |
| AT4G22140 | EBS | histone modification | [153] |
| AT4G39100 | SHL1 | histone modification | [153] |
| AT2G27880 | AGO5 | siRNA biogenesis | [154] |
| AT1G66170 | MMD1 | histone modification | [155] |
| AT3G03140 | PWO1 | histone modification | [156] |
| AT3G44750 | HDT1 | histone modification | [157] |
| AT5G22650 | HDT2 | histone modification | [157] |
| AT5G09230 | SRT2 | histone modification | [158] |
| AT5G55760 | SRT1 | histone modification | [158] |
| AT1G22950 | ICU11 | histone modification | [159] |
| AT3G18210 | CP2 | histone modification | [159] |
| AT1G08620 | PKDM7D | histone modification | [160] |
| AT3G15390 | SDE5 | siRNA biogenesis | [161] |
| AT1G16710 | HAC12 | histone modification | [162] |
| AT3G54610 | HAG1 | histone modification | [162] |
| AT5G56740 | HAG2 | histone modification | [162] |
| AT1G14400 | UBC1 | histone modification | [163] |
| AT2G02760 | UBC2 | histone modification | [163] |
| AT4G33470 | HDA14 | histone modification | [164] |
| AT1G73100 | SUVH3 | histone modification | [165] |
| AT2G24740 | SUVH8 | histone modification | [166] |
| AT3G12380 | ARP5 | chromatin remodeling | [167] |
| AT5G61060 | HDA05 | histone modification | [168] |
| AT1G09060 | JMJ24 | DNA methylation | [169] |
| AT1G32750 | HAF01 | histone modification | [170] |
| AT4G00990 | JMJ27 | histone modification | [171] |
| AT5G09740 | HAM2 | histone modification | [172] |
| AT5G64610 | HAM1 | histone modification | 170] |
| AT3G24870 | EAF1B | histone modification/ chromatin remodeling | [173] |
| AT3G24880 | EAF1A | histone modification/ chromatin remodeling | [173] |
| AT4G14385 | EAF6 | histone modification | [173] |
| AT1G57800 | VIM5 | DNA methylation | [53] |
| AT1G66040 | VIM4 | DNA methylation | [53] |
| AT3G04380 | SUVR4 | histone modification | [174] |
| AT1G08060 | MOM1 | chromatin remodeling? | [175] |
| AT1G30810 | JMJ18 | histone modification | [176] |
| AT2G23740 | SUVR5 | histone modification | [177] |
| AT1G14510 | ALF7 | histone modification | [178] |
| AT2G02470 | ALF6 | histone modification | [178] |
| AT5G05610 | ALF1 | histone modification | [178] |
| AT1G08460 | HDA08 | histone modification | [179] |
| AT3G63270 | ALP1 | histone modification | [180] |
| AT2G33290 | SUVH2 | histone modification | [181] |
| AT4G13460 | SUVH9 | histone modification | [181] |
| AT1G14790 | RDR1 | siRNA biogenesis | [182] |
| AT1G03770 | RING1B | histone modification | [183] |
| AT5G44280 | RING1A | histone modification | [183] |
| AT1G67220 | HAC2 | histone modification | [184] |
| AT1G80740 | CMT1 | DNA methylation | [185] |
| AT2G34900 | IMB1 | histone modification | [186] |

Reference

1. Pien, S.; Fleury, D.; Mylne, J.S.; Crevillen, P.; Inzé, D.; Avramova, Z.; Dean, C.; Grossniklaus, U. ARABIDOPSIS TRITHORAX1 Dynamically Regulates FLOWERING LOCUS C Activation via Histone 3 Lysine 4 Trimethylation. *Plant Cell* **2008**, *20*, 580–588, doi:10.1105/tpc.108.058172.

2. Derkacheva, M.; Steinbach, Y.; Wildhaber, T.; Mozgová, I.; Mahrez, W.; Nanni, P.; Bischof, S.; Gruissem, W.; Hennig, L. Arabidopsis MSI1 Connects LHP1 to PRC2 Complexes. *EMBO J.* **2013**, *32*, 2073–2085, doi:10.1038/emboj.2013.145.

3. Chen, H.; Chu, P.; Zhou, Y.; Li, Y.; Liu, J.; Ding, Y.; Tsang, E.W.T.; Jiang, L.; Wu, K.; Huang, S. Overexpression of AtOGG1, a DNA Glycosylase/AP Lyase, Enhances Seed Longevity and Abiotic Stress Tolerance in Arabidopsis. *J. Exp. Bot.* **2012**, *63*, 4107–4121, doi:10.1093/jxb/ers093.

4. Ebbs, M.L.; Bartee, L.; Bender, J. H3 Lysine 9 Methylation Is Maintained on a Transcribed Inverted Repeat by Combined Action of SUVH6 and SUVH4 Methyltransferases. *Mol. Cell. Biol.* **2005**, *25*, 10507–10515, doi:10.1128/MCB.25.23.10507-10515.2005.

5. Ortega-Galisteo, A.P.; Morales-Ruiz, T.; Ariza, R.R.; Roldán-Arjona, T. Arabidopsis DEMETER-LIKE Proteins DML2 and DML3 Are Required for Appropriate Distribution of DNA Methylation Marks. *Plant Mol. Biol.* **2008**, *67*, 671–681, doi:10.1007/s11103-008-9346-0.

6. He, X.-J.; Hsu, Y.-F.; Zhu, S.; Wierzbicki, A.T.; Pontes, O.; Pikaard, C.S.; Liu, H.-L.; Wang, C.-S.; Jin, H.; Zhu, J.-K. An Effector of RNA-Directed DNA Methylation in Arabidopsis Is an ARGONAUTE 4- and RNA-Binding Protein. *Cell* **2009**, *137*, 498–508, doi:10.1016/j.cell.2009.04.028.

7. Zemach, A.; Kim, M.Y.; Hsieh, P.-H.; Coleman-Derr, D.; Eshed-Williams, L.; Thao, K.; Harmer, S.L.; Zilberman, D. The Arabidopsis Nucleosome Remodeler DDM1 Allows DNA Methyltransferases to Access H1-Containing Heterochromatin. *Cell* **2013**, *153*, 193–205, doi:10.1016/j.cell.2013.02.033.

8. Kapoor, A.; Agarwal, M.; Andreucci, A.; Zheng, X.; Gong, Z.; Hasegawa, P.M.; Bressan, R.A.; Zhu, J.-K. Mutations in a Conserved Replication Protein Suppress Transcriptional Gene Silencing in a DNA- Methylation-Independent Manner in Arabidopsis. *Curr. Biol.* **2005**, *15*, 1912–1918, doi:10.1016/j.cub.2005.09.013.

9. Ream, T.S.; Haag, J.R.; Wierzbicki, A.T.; Nicora, C.D.; Norbeck, A.D.; Zhu, J.-K.; Hagen, G.; Guilfoyle, T.J.; Pasa-Tolić, L.; Pikaard, C.S. Subunit Compositions of the RNA-Silencing Enzymes Pol IV and Pol V Reveal Their Origins as Specialized Forms of RNA Polymerase II. *Mol. Cell* **2009**, *33*, 192–203, doi:10.1016/j.molcel.2008.12.015.

10. Du, J.; Johnson, L.M.; Groth, M.; Feng, S.; Hale, C.J.; Li, S.; Vashisht, A.A.; Gallego-Bartolome, J.; Wohlschlegel, J.A.; Patel, D.J.; et al. Mechanism of DNA Methylation-Directed Histone Methylation by KRYPTONITE. *Mol. Cell* **2014**, *55*, 495–504, doi:10.1016/j.molcel.2014.06.009.

11. Zemach, A.; Grafi, G. Methyl-CpG-Binding Domain Proteins in Plants: Interpreters of DNA Methylation. *Trends Plant Sci.* **2007**, *12*, 80–85, doi:10.1016/j.tplants.2006.12.004.

12. Skylar, A.; Matsuwaka, S.; Wu, X. ELONGATA3 Is Required for Shoot Meristem Cell Cycle Progression in Arabidopsis Thaliana Seedlings. *Dev. Biol.* **2013**, *382*, 436–445, doi:10.1016/j.ydbio.2013.08.008.

13. Qi, Y.; He, X.; Wang, X.-J.; Kohany, O.; Jurka, J.; Hannon, G.J. Distinct Catalytic and Non-Catalytic Roles of ARGONAUTE4 in RNA-Directed DNA Methylation. *Nature* **2006**, *443*, 1008–1012, doi:10.1038/nature05198.

14. Olmedo-Monfil, V.; Durán-Figueroa, N.; Arteaga-Vázquez, M.; Demesa-Arévalo, E.; Autran, D.; Grimanelli, D.; Slotkin, R.K.; Martienssen, R.A.; Vielle-Calzada, J.-P. Control of Female Gamete Formation by a Small RNA Pathway in Arabidopsis. *Nature* **2010**, *464*, 628–632, doi:10.1038/nature08828.

15. Fong, P.M.; Tian, L.; Chen, Z.J. Arabidopsis Thaliana Histone Deacetylase 1 (AtHD1) Is Localized in Euchromatic Regions and Demonstrates Histone Deacetylase Activity in Vitro. *Cell Res.* **2006**, *16*, 479–488, doi:10.1038/sj.cr.7310059.

16. Yan, D.; Zhang, Y.; Niu, L.; Yuan, Y.; Cao, X. Identification and Characterization of Two Closely Related Histone H4 Arginine 3 Methyltransferases in Arabidopsis Thaliana. *Biochem. J.* **2007**, *408*, 113–121, doi:10.1042/BJ20070786.

17. Yoshikawa, M.; Iki, T.; Tsutsui, Y.; Miyashita, K.; Poethig, R.S.; Habu, Y.; Ishikawa, M. 3’ Fragment of MiR173-Programmed RISC-Cleaved RNA Is Protected from Degradation in a Complex with RISC and SGS3. *Proc. Natl. Acad. Sci.* **2013**, *110*, 4117–4122, doi:10.1073/pnas.1217050110.

18. Hang, R.; Liu, C.; Ahmad, A.; Zhang, Y.; Lu, F.; Cao, X. *Arabidopsis* Protein Arginine Methyltransferase 3 Is Required for Ribosome Biogenesis by Affecting Precursor Ribosomal RNA Processing. *Proc. Natl. Acad. Sci.* **2014**, *111*, 16190–16195, doi:10.1073/pnas.1412697111.

19. Groth, M.; Stroud, H.; Feng, S.; Greenberg, M.V.C.; Vashisht, A.A.; Wohlschlegel, J.A.; Jacobsen, S.E.; Ausin, I. SNF2 Chromatin Remodeler-Family Proteins FRG1 and -2 Are Required for RNA-Directed DNA Methylation. *Proc. Natl. Acad. Sci.* **2014**, *111*, 17666–17671, doi:10.1073/pnas.1420515111.

20. Martínez-Macías, M.I.; Córdoba-Cañero, D.; Ariza, R.R.; Roldán-Arjona, T. The DNA Repair Protein XRCC1 Functions in the Plant DNA Demethylation Pathway by Stimulating Cytosine Methylation (5-MeC) Excision, Gap Tailoring, and DNA Ligation. *J. Biol. Chem.* **2013**, *288*, 5496–5505, doi:10.1074/jbc.M112.427617.

21. Bertrand, C.; Benhamed, M.; Li, Y.-F.; Ayadi, M.; Lemonnier, G.; Renou, J.-P.; Delarue, M.; Zhou, D.-X. *Arabidopsis* HAF2 Gene Encoding TATA-Binding Protein (TBP)-Associated Factor TAF1, Is Required to Integrate Light Signals to Regulate Gene Expression and Growth. *J. Biol. Chem.* **2005**, *280*, 1465–1473, doi:10.1074/jbc.M409000200.

22. Uddin, M.N.; Dunoyer, P.; Schott, G.; Akhter, S.; Shi, C.; Lucas, W.J.; Voinnet, O.; Kim, J.-Y. The Protein Kinase TOUSLED Facilitates RNAi in *Arabidopsis*. *Nucleic Acids Res.* **2014**, *42*, 7971–7980, doi:10.1093/nar/gku422.

23. Xu, L.; Yang, L.; Pi, L.; Liu, Q.; Ling, Q.; Wang, H.; Poethig, R.S.; Huang, H. Genetic Interaction between the AS1–AS2 and RDR6–SGS3–AGO7 Pathways for Leaf Morphogenesis. *Plant Cell Physiol.* **2006**, *47*, 853–863, doi:10.1093/pcp/pcj057.

24. Rajakumara, E.; Law, J.A.; Simanshu, D.K.; Voigt, P.; Johnson, L.M.; Reinberg, D.; Patel, D.J.; Jacobsen, S.E. A Dual Flip-out Mechanism for 5mC Recognition by the Arabidopsis SUVH5 SRA Domain and Its Impact on DNA Methylation and H3K9 Dimethylation in Vivo. *Genes Dev.* **2011**, *25*, 137–152, doi:10.1101/gad.1980311.

25. Li, S.; Vandivier, L.E.; Tu, B.; Gao, L.; Won, S.Y.; Li, S.; Zheng, B.; Gregory, B.D.; Chen, X. Detection of Pol IV/RDR2-Dependent Transcripts at the Genomic Scale in *Arabidopsis* Reveals Features and Regulation of SiRNA Biogenesis. *Genome Res.* **2015**, *25*, 235–245, doi:10.1101/gr.182238.114.

26. Sarnowski, T.J.; Ríos, G.; Jásik, J.; Świeżewski, S.; Kaczanowski, S.; Li, Y.; Kwiatkowska, A.; Pawlikowska, K.; Koźbiał, M.; Koźbiał, P.; et al. SWI3 Subunits of Putative SWI/SNF Chromatin-Remodeling Complexes Play Distinct Roles during *Arabidopsis* Development. *Plant Cell* **2005**, *17*, 2454–2472, doi:10.1105/tpc.105.031203.

27. Bezhani, S.; Winter, C.; Hershman, S.; Wagner, J.D.; Kennedy, J.F.; Kwon, C.S.; Pfluger, J.; Su, Y.; Wagner, D. Unique, Shared, and Redundant Roles for the Arabidopsis SWI/SNF Chromatin Remodeling ATPases BRAHMA and SPLAYED. *Plant Cell* **2007**, *19*, 403–416, doi:10.1105/tpc.106.048272.

28. Smith, L.M.; Pontes, O.; Searle, I.; Yelina, N.; Yousafzai, F.K.; Herr, A.J.; Pikaard, C.S.; Baulcombe, D.C. An SNF2 Protein Associated with Nuclear RNA Silencing and the Spread of a Silencing Signal between Cells in *Arabidopsis*. *Plant Cell* **2007**, *19*, 1507–1521, doi:10.1105/tpc.107.051540.

29. Li, H.; Soriano, M.; Cordewener, J.; Muiño, J.M.; Riksen, T.; Fukuoka, H.; Angenent, G.C.; Boutilier, K. The Histone Deacetylase Inhibitor Trichostatin A Promotes Totipotency in the Male Gametophyte. *Plant Cell* **2014**, *26*, 195–209, doi:10.1105/tpc.113.116491.

30. Kandasamy, M.K.; Deal, R.B.; McKinney, E.C.; Meagher, R.B. Silencing the Nuclear Actin-Related Protein AtARP4 in Arabidopsis Has Multiple Effects on Plant Development, Including Early Flowering and Delayed Floral Senescence: Role of AtARP4 in Plant Development. *Plant J.* **2005**, *41*, 845–858, doi:10.1111/j.1365-313X.2005.02345.x.

31. Mlynárová, L.; Nap, J.-P.; Bisseling, T. The SWI/SNF Chromatin-Remodeling Gene AtCHR12 Mediates Temporary Growth Arrest in Arabidopsis Thaliana upon Perceiving Environmental Stress. *Plant J.* **2007**, *51*, 874–885, doi:10.1111/j.1365-313X.2007.03185.x.

32. Shen, Y.; Devic, M.; Lepiniec, L.; Zhou, D.-X. Chromodomain, Helicase and DNA-Binding CHD1 Protein, CHR5, Are Involved in Establishing Active Chromatin State of Seed Maturation Genes. *Plant Biotechnol. J.* **2015**, *13*, 811–820, doi:10.1111/pbi.12315.

33. Fan, H.; Zhang, Z.; Wang, N.; Cui, Y.; Sun, H.; Liu, Y.; Wu, H.; Zheng, S.; Bao, S.; Ling, H.-Q. SKB1/PRMT5-Mediated Histone H4R3 Dimethylation of Ib Subgroup BHLH Genes Negatively Regulates Iron Homeostasis in Arabidopsis Thaliana. *Plant J.* **2014**, *77*, 209–221, doi:10.1111/tpj.12380.

34. Li, G.; Liu, S.; Wang, J.; He, J.; Huang, H.; Zhang, Y.; Xu, L. ISWI Proteins Participate in the Genome-Wide Nucleosome Distribution in Arabidopsis. *Plant J.* **2014**, *78*, 706–714, doi:10.1111/tpj.12499.

35. Parent, J.-S.; Bouteiller, N.; Elmayan, T.; Vaucheret, H. Respective Contributions of Arabidopsis DCL2 and DCL4 to RNA Silencing. *Plant J.* **2015**, *81*, 223–232, doi:10.1111/tpj.12720.

36. Lindroth, A.M. Requirement of CHROMOMETHYLASE3 for Maintenance of CpXpG Methylation. *Science* **2001**, *292*, 2077–2080, doi:10.1126/science.1059745.

37. He, Y. Regulation of Flowering Time by Histone Acetylation in Arabidopsis. *Science* **2003**, *302*, 1751–1754, doi:10.1126/science.1091109.

38. Herr, A.J. RNA Polymerase IV Directs Silencing of Endogenous DNA. *Science* **2005**, *308*, 118–120, doi:10.1126/science.1106910.

39. Baurle, I.; Smith, L.; Baulcombe, D.C.; Dean, C. Widespread Role for the Flowering-Time Regulators FCA and FPA in RNA-Mediated Chromatin Silencing. *Science* **2007**, *318*, 109–112, doi:10.1126/science.1146565.

40. Saze, H.; Shiraishi, A.; Miura, A.; Kakutani, T. Control of Genic DNA Methylation by a JmjC Domain-Containing Protein in Arabidopsis Thaliana. *Science* **2008**, *319*, 462–465, doi:10.1126/science.1150987.

41. Qian, W.; Miki, D.; Zhang, H.; Liu, Y.; Zhang, X.; Tang, K.; Kan, Y.; La, H.; Li, X.; Li, S.; et al. A Histone Acetyltransferase Regulates Active DNA Demethylation in Arabidopsis. *Science* **2012**, *336*, 1445–1448, doi:10.1126/science.1219416.

42. Moissiard, G.; Cokus, S.J.; Cary, J.; Feng, S.; Billi, A.C.; Stroud, H.; Husmann, D.; Zhan, Y.; Lajoie, B.R.; McCord, R.P.; et al. MORC Family ATPases Required for Heterochromatin Condensation and Gene Silencing. *Science* **2012**, *336*, 1448–1451, doi:10.1126/science.1221472.

43. Grossniklaus, U. Maternal Control of Embryogenesis by MEDEA, a Polycomb Group Gene in Arabidopsis. *Science* **1998**, *280*, 446–450, doi:10.1126/science.280.5362.446.

44. Lee, K.; Park, O.-S.; Seo, P.J. *Arabidopsis* ATXR2 Deposits H3K36me3 at the Promoters of *LBD* Genes to Facilitate Cellular Dedifferentiation. *Sci. Signal.* **2017**, *10*, eaan0316, doi:10.1126/scisignal.aan0316.

45. Li, Y.; Mukherjee, I.; Thum, K.E.; Tanurdzic, M.; Katari, M.S.; Obertello, M.; Edwards, M.B.; McCombie, W.R.; Martienssen, R.A.; Coruzzi, G.M. The Histone Methyltransferase SDG8 Mediates the Epigenetic Modification of Light and Carbon Responsive Genes in Plants. *Genome Biol.* **2015**, *16*, 79, doi:10.1186/s13059-015-0640-2.

46. Folta, A.; Severing, E.I.; Krauskopf, J.; van de Geest, H.; Verver, J.; Nap, J.-P.; Mlynarova, L. Over-Expression of Arabidopsis AtCHR23 Chromatin Remodeling ATPase Results in Increased Variability of Growth and Gene Expression. *BMC Plant Biol.* **2014**, *14*, 76, doi:10.1186/1471-2229-14-76.

47. Hernando, C.E.; Sanchez, S.E.; Mancini, E.; Yanovsky, M.J. Genome Wide Comparative Analysis of the Effects of PRMT5 and PRMT4/CARM1 Arginine Methyltransferases on the Arabidopsis Thaliana Transcriptome. *BMC Genomics* **2015**, *16*, doi:10.1186/s12864-015-1399-2.

48. Roodbarkelari, F.; Du, F.; Truernit, E.; Laux, T. ZLL/AGO10 Maintains Shoot Meristem Stem Cells during Arabidopsis Embryogenesis by down-Regulating ARF2-Mediated Auxin Response. *BMC Biol.* **2015**, *13*, doi:10.1186/s12915-015-0180-y.

49. Choi, K.; Park, C.; Lee, J.; Oh, M.; Noh, B.; Lee, I. Arabidopsis Homologs of Components of the SWR1 Complex Regulate Flowering and Plant Development. *Development* **2007**, *134*, 1931–1941, doi:10.1242/dev.001891.

50. Farrona, S. The Arabidopsis Thaliana SNF2 Homolog AtBRM Controls Shoot Development and Flowering. *Development* **2004**, *131*, 4965–4975, doi:10.1242/dev.01363.

51. Exner, V.; Taranto, P.; Schonrock, N.; Gruissem, W.; Hennig, L. Chromatin Assembly Factor CAF-1 Is Required for Cellular Differentiation during Plant Development. *Development* **2006**, *133*, 4163–4172, doi:10.1242/dev.02599.

52. Kurihara, Y. The Interaction between DCL1 and HYL1 Is Important for Efficient and Precise Processing of Pri-MiRNA in Plant MicroRNA Biogenesis. *RNA* **2005**, *12*, 206–212, doi:10.1261/rna.2146906.

53. Woo, H.R.; Dittmer, T.A.; Richards, E.J. Three SRA-Domain Methylcytosine-Binding Proteins Cooperate to Maintain Global CpG Methylation and Epigenetic Silencing in Arabidopsis. *PLoS Genet.* **2008**, *4*, e1000156, doi:10.1371/journal.pgen.1000156.

54. Henderson, I.R.; Deleris, A.; Wong, W.; Zhong, X.; Chin, H.G.; Horwitz, G.A.; Kelly, K.A.; Pradhan, S.; Jacobsen, S.E. The De Novo Cytosine Methyltransferase DRM2 Requires Intact UBA Domains and a Catalytically Mutated Paralog DRM3 during RNA–Directed DNA Methylation in Arabidopsis Thaliana. *PLoS Genet.* **2010**, *6*, e1001182, doi:10.1371/journal.pgen.1001182.

55. Bouyer, D.; Roudier, F.; Heese, M.; Andersen, E.D.; Gey, D.; Nowack, M.K.; Goodrich, J.; Renou, J.-P.; Grini, P.E.; Colot, V.; et al. Polycomb Repressive Complex 2 Controls the Embryo-to-Seedling Phase Transition. *PLoS Genet.* **2011**, *7*, e1002014, doi:10.1371/journal.pgen.1002014.

56. Law, J.A.; Vashisht, A.A.; Wohlschlegel, J.A.; Jacobsen, S.E. SHH1, a Homeodomain Protein Required for DNA Methylation, As Well As RDR2, RDM4, and Chromatin Remodeling Factors, Associate with RNA Polymerase IV. *PLoS Genet.* **2011**, *7*, e1002195, doi:10.1371/journal.pgen.1002195.

57. Gu, X.; Jiang, D.; Yang, W.; Jacob, Y.; Michaels, S.D.; He, Y. Arabidopsis Homologs of Retinoblastoma-Associated Protein 46/48 Associate with a Histone Deacetylase to Act Redundantly in Chromatin Silencing. *PLoS Genet.* **2011**, *7*, e1002366, doi:10.1371/journal.pgen.1002366.

58. Kim, S.Y.; Lee, J.; Eshed-Williams, L.; Zilberman, D.; Sung, Z.R. EMF1 and PRC2 Cooperate to Repress Key Regulators of Arabidopsis Development. *PLoS Genet.* **2012**, *8*, e1002512, doi:10.1371/journal.pgen.1002512.

59. Duan, C.-G.; Wang, X.; Tang, K.; Zhang, H.; Mangrauthia, S.K.; Lei, M.; Hsu, C.-C.; Hou, Y.-J.; Wang, C.; Li, Y.; et al. MET18 Connects the Cytosolic Iron-Sulfur Cluster Assembly Pathway to Active DNA Demethylation in Arabidopsis. *PLOS Genet.* **2015**, *11*, e1005559, doi:10.1371/journal.pgen.1005559.

60. Harvey, J.J.W.; Lewsey, M.G.; Patel, K.; Westwood, J.; Heimstädt, S.; Carr, J.P.; Baulcombe, D.C. An Antiviral Defense Role of AGO2 in Plants. *PLoS ONE* **2011**, *6*, e14639, doi:10.1371/journal.pone.0014639.

61. Duan, C.; Zhang, H.; Tang, K.; Zhu, X.; Qian, W.; Hou, Y.; Wang, B.; Lang, Z.; Zhao, Y.; Wang, X.; et al. Specific but Interdependent Functions for  *A Rabidopsis*  AGO 4 and AGO 6 in RNA ‐directed DNA Methylation. *EMBO J.* **2015**, *34*, 581–592, doi:10.15252/embj.201489453.

62. Schmitz, R.J.; Hong, L.; Fitzpatrick, K.E.; Amasino, R.M. *DICER-LIKE 1* and *DICER-LIKE 3* Redundantly Act to Promote Flowering via Repression of *FLOWERING LOCUS C* in *Arabidopsis Thaliana*. *Genetics* **2007**, *176*, 1359–1362, doi:10.1534/genetics.107.070649.

63. Meagher, R.B.; Kandasamy, M.K.; Smith, A.P.; McKinney, E.C. Nuclear Actin-Related Proteins at the Core of Epigenetic Control. *Plant Signal. Behav.* **2010**, *5*, 518–522, doi:10.4161/psb.10986.

64. Li, C.; Xu, J.; Li, J.; Li, Q.; Yang, H. Involvement of *Arabidopsis HAC* Family Genes in Pleiotropic Developmental Processes. *Plant Signal. Behav.* **2014**, *9*, e28173, doi:10.4161/psb.28173.

65. Niu, L.; Lu, F.; Pei, Y.; Liu, C.; Cao, X. Regulation of Flowering Time by the Protein Arginine Methyltransferase AtPRMT10. *EMBO Rep.* **2007**, *8*, 1190–1195, doi:10.1038/sj.embor.7401111.

66. Garcia-Dominguez, M.; March-Diaz, R.; Reyes, J.C. The PHD Domain of Plant PIAS Proteins Mediates Sumoylation of Bromodomain GTE Proteins. *J. Biol. Chem.* **2008**, *283*, 21469–21477, doi:10.1074/jbc.M708176200.

67. To, T.K.; Nakaminami, K.; Kim, J.-M.; Morosawa, T.; Ishida, J.; Tanaka, M.; Yokoyama, S.; Shinozaki, K.; Seki, M. Arabidopsis HDA6 Is Required for Freezing Tolerance. *Biochem. Biophys. Res. Commun.* **2011**, *406*, 414–419, doi:10.1016/j.bbrc.2011.02.058.

68. Yang, H.; Mo, H.; Fan, D.; Cao, Y.; Cui, S.; Ma, L. Overexpression of a Histone H3K4 Demethylase, JMJ15, Accelerates Flowering Time in Arabidopsis. *Plant Cell Rep.* **2012**, *31*, 1297–1308, doi:10.1007/s00299-012-1249-5.

69. Liang, X.; Lei, M.; Li, F.; Yang, X.; Zhou, M.; Li, B.; Cao, Y.; Gong, S.; Liu, K.; Liu, J.; et al. Family-Wide Characterization of Histone Binding Abilities of PHD Domains of AL Proteins in Arabidopsis Thaliana. *Protein J.* **2018**, *37*, 531–538, doi:10.1007/s10930-018-9796-4.

70. Misra, A.; McKnight, T.D.; Mandadi, K.K. Bromodomain Proteins GTE9 and GTE11 Are Essential for Specific BT2-Mediated Sugar and ABA Responses in Arabidopsis Thaliana. *Plant Mol. Biol.* **2018**, *96*, 393–402, doi:10.1007/s11103-018-0704-2.

71. Houben, A.; Demidov, D.; Caperta, A.D.; Karimi, R.; Agueci, F.; Vlasenko, L. Phosphorylation of Histone H3 in Plants—A Dynamic Affair. *Biochim. Biophys. Acta BBA - Gene Struct. Expr.* **2007**, *1769*, 308–315, doi:10.1016/j.bbaexp.2007.01.002.

72. Ng, D.W.-K.; Wang, T.; Chandrasekharan, M.B.; Aramayo, R.; Kertbundit, S.; Hall, T.C. Plant SET Domain-Containing Proteins: Structure, Function and Regulation. *Biochim. Biophys. Acta* **2007**, *1769*, 316–329, doi:10.1016/j.bbaexp.2007.04.003.

73. Chen, X.; Hu, Y.; Zhou, D.-X. Epigenetic Gene Regulation by Plant Jumonji Group of Histone Demethylase. *Biochim. Biophys. Acta BBA - Gene Regul. Mech.* **2011**, *1809*, 421–426, doi:10.1016/j.bbagrm.2011.03.004.

74. Song, Y.; Wu, K.; Dhaubhadel, S.; An, L.; Tian, L. Arabidopsis DNA Methyltransferase AtDNMT2 Associates with Histone Deacetylase AtHD2s Activity. *Biochem. Biophys. Res. Commun.* **2010**, *396*, 187–192, doi:10.1016/j.bbrc.2010.03.119.

75. Wierzbicki, A.T.; Haag, J.R.; Pikaard, C.S. Noncoding Transcription by RNA Polymerase Pol IVb/Pol V Mediates Transcriptional Silencing of Overlapping and Adjacent Genes. *Cell* **2008**, *135*, 635–648, doi:10.1016/j.cell.2008.09.035.

76. Bratzel, F.; López-Torrejón, G.; Koch, M.; Del Pozo, J.C.; Calonje, M. Keeping Cell Identity in Arabidopsis Requires PRC1 RING-Finger Homologs That Catalyze H2A Monoubiquitination. *Curr. Biol.* **2010**, *20*, 1853–1859, doi:10.1016/j.cub.2010.09.046.

77. Liu, C.; Xin, Y.; Xu, L.; Cai, Z.; Xue, Y.; Liu, Y.; Xie, D.; Liu, Y.; Qi, Y. Arabidopsis ARGONAUTE 1 Binds Chromatin to Promote Gene Transcription in Response to Hormones and Stresses. *Dev. Cell* **2018**, *44*, 348-361.e7, doi:10.1016/j.devcel.2017.12.002.

78. Fritsch, O.; Benvenuto, G.; Bowler, C.; Molinier, J.; Hohn, B. The INO80 Protein Controls Homologous Recombination in Arabidopsis Thaliana. *Mol. Cell* **2004**, *16*, 479–485, doi:10.1016/j.molcel.2004.09.034.

79. Garcia, D.; Garcia, S.; Pontier, D.; Marchais, A.; Renou, J.P.; Lagrange, T.; Voinnet, O. Ago Hook and RNA Helicase Motifs Underpin Dual Roles for SDE3 in Antiviral Defense and Silencing of Nonconserved Intergenic Regions. *Mol. Cell* **2012**, *48*, 109–120, doi:10.1016/j.molcel.2012.07.028.

80. Qian, W.; Miki, D.; Lei, M.; Zhu, X.; Zhang, H.; Liu, Y.; Li, Y.; Lang, Z.; Wang, J.; Tang, K.; et al. Regulation of Active DNA Demethylation by an α-Crystallin Domain Protein in Arabidopsis. *Mol. Cell* **2014**, *55*, 361–371, doi:10.1016/j.molcel.2014.06.008.

81. Lang, Z.; Lei, M.; Wang, X.; Tang, K.; Miki, D.; Zhang, H.; Mangrauthia, S.K.; Liu, W.; Nie, W.; Ma, G.; et al. The Methyl-CpG-Binding Protein MBD7 Facilitates Active DNA Demethylation to Limit DNA Hyper-Methylation and Transcriptional Gene Silencing. *Mol. Cell* **2015**, *57*, 971–983, doi:10.1016/j.molcel.2015.01.009.

82. Zhang, H.; Xia, R.; Meyers, B.C.; Walbot, V. Evolution, Functions, and Mysteries of Plant ARGONAUTE Proteins. *Curr. Opin. Plant Biol.* **2015**, *27*, 84–90, doi:10.1016/j.pbi.2015.06.011.

83. Krause, C.D.; Yang, Z.-H.; Kim, Y.-S.; Lee, J.-H.; Cook, J.R.; Pestka, S. Protein Arginine Methyltransferases: Evolution and Assessment of Their Pharmacological and Therapeutic Potential. *Pharmacol. Ther.* **2007**, *113*, 50–87, doi:10.1016/j.pharmthera.2006.06.007.

84. Cartagena, J.A.; Matsunaga, S.; Seki, M.; Kurihara, D.; Yokoyama, M.; Shinozaki, K.; Fujimoto, S.; Azumi, Y.; Uchiyama, S.; Fukui, K. The Arabidopsis SDG4 Contributes to the Regulation of Pollen Tube Growth by Methylation of Histone H3 Lysines 4 and 36 in Mature Pollen. *Dev. Biol.* **2008**, *315*, 355–368, doi:10.1016/j.ydbio.2007.12.016.

85. Gendall, A.R.; Levy, Y.Y.; Wilson, A.; Dean, C. The VERNALIZATION 2 Gene Mediates the Epigenetic Regulation of Vernalization in Arabidopsis. *Cell* **2001**, *107*, 525–535, doi:10.1016/S0092-8674(01)00573-6.

86. Choi, Y.; Gehring, M.; Johnson, L.; Hannon, M.; Harada, J.J.; Goldberg, R.B.; Jacobsen, S.E.; Fischer, R.L. DEMETER, a DNA Glycosylase Domain Protein, Is Required for Endosperm Gene Imprinting and Seed Viability in Arabidopsis. *Cell* **2002**, *110*, 33–42, doi:10.1016/S0092-8674(02)00807-3.

87. Gong, Z.; Morales-Ruiz, T.; Ariza, R.R.; Roldán-Arjona, T.; David, L.; Zhu, J.-K. ROS1, a Repressor of Transcriptional Gene Silencing in Arabidopsis, Encodes a DNA Glycosylase/Lyase. *Cell* **2002**, *111*, 803–814, doi:10.1016/S0092-8674(02)01133-9.

88. Jones, L.; Ratcliff, F.; Baulcombe, D.C. RNA-Directed Transcriptional Gene Silencing in Plants Can Be Inherited Independently of the RNA Trigger and Requires Met1 for Maintenance. *Curr. Biol.* **2001**, *11*, 747–757, doi:10.1016/S0960-9822(01)00226-3.

89. Cao, X.; Jacobsen, S.E. Role of the Arabidopsis DRM Methyltransferases in De Novo DNA Methylation and Gene Silencing. *Curr. Biol.* **2002**, *12*, 1138–1144, doi:10.1016/S0960-9822(02)00925-9.

90. Boutet, S.; Vazquez, F.; Liu, J.; Béclin, C.; Fagard, M.; Gratias, A.; Morel, J.-B.; Crété, P.; Chen, X.; Vaucheret, H. Arabidopsis HEN1. *Curr. Biol.* **2003**, *13*, 843–848, doi:10.1016/S0960-9822(03)00293-8.

91. Genger, R.K.; Kovac, K.A.; Dennis, E.S.; Peacock, W.J.; Finnegan, E.J. [No Title Found]. *Plant Mol. Biol.* **1999**, *41*, 269–278, doi:10.1023/A:1006347010369.

92. Goodrich, J.; Puangsomlee, P.; Martin, M.; Long, D.; Meyerowitz, E.M.; Coupland, G. A Polycomb-Group Gene Regulates Homeotic Gene Expression in Arabidopsis. *Nature* **1997**, *386*, 44–51, doi:10.1038/386044a0.

93. Lu, F.; Cui, X.; Zhang, S.; Liu, C.; Cao, X. JMJ14 Is an H3K4 Demethylase Regulating Flowering Time in Arabidopsis. *Cell Res.* **2010**, *20*, 387–390, doi:10.1038/cr.2010.27.

94. Han, Y.-F.; Dou, K.; Ma, Z.-Y.; Zhang, S.-W.; Huang, H.-W.; Li, L.; Cai, T.; Chen, S.; Zhu, J.-K.; He, X.-J. SUVR2 Is Involved in Transcriptional Gene Silencing by Associating with SNF2-Related Chromatin-Remodeling Proteins in Arabidopsis. *Cell Res.* **2014**, *24*, 1445–1465, doi:10.1038/cr.2014.156.

95. Duan, C.-G.; Wang, X.; Xie, S.; Pan, L.; Miki, D.; Tang, K.; Hsu, C.-C.; Lei, M.; Zhong, Y.; Hou, Y.-J.; et al. A Pair of Transposon-Derived Proteins Function in a Histone Acetyltransferase Complex for Active DNA Demethylation. *Cell Res.* **2017**, *27*, 226–240, doi:10.1038/cr.2016.147.

96. Jouannet, V.; Moreno, A.B.; Elmayan, T.; Vaucheret, H.; Crespi, M.D.; Maizel, A. Cytoplasmic Arabidopsis AGO7 Accumulates in Membrane-Associated SiRNA Bodies and Is Required for Ta-SiRNA Biogenesis: AGO7 Associates with Membranes. *EMBO J.* **2012**, *31*, 1704–1713, doi:10.1038/emboj.2012.20.

97. Zheng, X.; Pontes, O.; Zhu, J.; Miki, D.; Zhang, F.; Li, W.-X.; Iida, K.; Kapoor, A.; Pikaard, C.S.; Zhu, J.-K. ROS3 Is an RNA-Binding Protein Required for DNA Demethylation in Arabidopsis. *Nature* **2008**, *455*, 1259–1262, doi:10.1038/nature07305.

98. Gao, Z.; Liu, H.-L.; Daxinger, L.; Pontes, O.; He, X.; Qian, W.; Lin, H.; Xie, M.; Lorkovic, Z.J.; Zhang, S.; et al. An RNA Polymerase II- and AGO4-Associated Protein Acts in RNA-Directed DNA Methylation. *Nature* **2010**, *465*, 106–109, doi:10.1038/nature09025.

99. Crevillén, P.; Yang, H.; Cui, X.; Greeff, C.; Trick, M.; Qiu, Q.; Cao, X.; Dean, C. Epigenetic Reprogramming That Prevents Transgenerational Inheritance of the Vernalized State. *Nature* **2014**, *515*, 587–590, doi:10.1038/nature13722.

100. Zhang, C.-J.; Hou, X.-M.; Tan, L.-M.; Shao, C.-R.; Huang, H.-W.; Li, Y.-Q.; Li, L.; Cai, T.; Chen, S.; He, X.-J. The Arabidopsis Acetylated Histone-Binding Protein BRAT1 Forms a Complex with BRP1 and Prevents Transcriptional Silencing. *Nat. Commun.* **2016**, *7*, doi:10.1038/ncomms11715.

101. Lu, F.; Cui, X.; Zhang, S.; Jenuwein, T.; Cao, X. Arabidopsis REF6 Is a Histone H3 Lysine 27 Demethylase. *Nat. Genet.* **2011**, *43*, 715–719, doi:10.1038/ng.854.

102. Derkacheva, M.; Liu, S.; Figueiredo, D.D.; Gentry, M.; Mozgova, I.; Nanni, P.; Tang, M.; Mannervik, M.; Köhler, C.; Hennig, L. H2A Deubiquitinases UBP12/13 Are Part of the Arabidopsis Polycomb Group Protein System. *Nat. Plants* **2016**, *2*, doi:10.1038/nplants.2016.126.

103. Zhang, Z.; Liu, X.; Guo, X.; Wang, X.-J.; Zhang, X. Arabidopsis AGO3 Predominantly Recruits 24-Nt Small RNAs to Regulate Epigenetic Silencing. *Nat. Plants* **2016**, *2*, doi:10.1038/nplants.2016.49.

104. Jacob, Y.; Feng, S.; LeBlanc, C.A.; Bernatavichute, Y.V.; Stroud, H.; Cokus, S.; Johnson, L.M.; Pellegrini, M.; Jacobsen, S.E.; Michaels, S.D. ATXR5 and ATXR6 Are H3K27 Monomethyltransferases Required for Chromatin Structure and Gene Silencing. *Nat. Struct. Mol. Biol.* **2009**, *16*, 763–768, doi:10.1038/nsmb.1611.

105. Ausin, I.; Mockler, T.C.; Chory, J.; Jacobsen, S.E. IDN1 and IDN2 Are Required for de Novo DNA Methylation in Arabidopsis Thaliana. *Nat. Struct. Mol. Biol.* **2009**, *16*, 1325–1327, doi:10.1038/nsmb.1690.

106. Zhao, S.; Cheng, L.; Gao, Y.; Zhang, B.; Zheng, X.; Wang, L.; Li, P.; Sun, Q.; Li, H. Plant HP1 Protein ADCP1 Links Multivalent H3K9 Methylation Readout to Heterochromatin Formation. *Cell Res.* **2019**, *29*, 54–66, doi:10.1038/s41422-018-0104-9.

107. Zheng, S.; Hu, H.; Ren, H.; Yang, Z.; Qiu, Q.; Qi, W.; Liu, X.; Chen, X.; Cui, X.; Li, S.; et al. The Arabidopsis H3K27me3 Demethylase JUMONJI 13 Is a Temperature and Photoperiod Dependent Flowering Repressor. *Nat. Commun.* **2019**, *10*, doi:10.1038/s41467-019-09310-x.

108. Baumberger, N.; Baulcombe, D.C. Arabidopsis ARGONAUTE1 Is an RNA Slicer That Selectively Recruits MicroRNAs and Short Interfering RNAs. *Proc. Natl. Acad. Sci.* **2005**, *102*, 11928–11933, doi:10.1073/pnas.0505461102.

109. Huanca-Mamani, W.; Garcia-Aguilar, M.; Leon-Martinez, G.; Grossniklaus, U.; Vielle-Calzada, J.-P. CHR11, a Chromatin-Remodeling Factor Essential for Nuclear Proliferation during Female Gametogenesis in Arabidopsis Thaliana. *Proc. Natl. Acad. Sci.* **2005**, *102*, 17231–17236, doi:10.1073/pnas.0508186102.

110. de la Paz Sanchez, M.; Gutierrez, C. *Arabidopsis* ORC1 Is a PHD-Containing H3K4me3 Effector That Regulates Transcription. *Proc. Natl. Acad. Sci.* **2009**, *106*, 2065–2070, doi:10.1073/pnas.0811093106.

111. Guo, L.; Yu, Y.; Law, J.A.; Zhang, X. SET DOMAIN GROUP2 Is the Major Histone H3 Lysine 4 Trimethyltransferase in Arabidopsis. *Proc. Natl. Acad. Sci.* **2010**, *107*, 18557–18562, doi:10.1073/pnas.1010478107.

112. Krichevsky, A.; Zaltsman, A.; Lacroix, B.; Citovsky, V. Involvement of KDM1C Histone Demethylase–OTLD1 Otubain-like Histone Deubiquitinase Complexes in Plant Gene Repression. *Proc. Natl. Acad. Sci.* **2011**, *108*, 11157–11162, doi:10.1073/pnas.1014030108.

113. Jones, M.A.; Covington, M.F.; DiTacchio, L.; Vollmers, C.; Panda, S.; Harmer, S.L. Jumonji Domain Protein JMJD5 Functions in Both the Plant and Human Circadian Systems. *Proc. Natl. Acad. Sci.* **2010**, *107*, 21623–21628, doi:10.1073/pnas.1014204108.

114. Wang, X.; Duan, C.-G.; Tang, K.; Wang, B.; Zhang, H.; Lei, M.; Lu, K.; Mangrauthia, S.K.; Wang, P.; Zhu, G.; et al. RNA-Binding Protein Regulates Plant DNA Methylation by Controlling MRNA Processing at the Intronic Heterochromatin-Containing Gene IBM1. *Proc. Natl. Acad. Sci.* **2013**, *110*, 15467–15472, doi:10.1073/pnas.1315399110.

115. Lei, M.; La, H.; Lu, K.; Wang, P.; Miki, D.; Ren, Z.; Duan, C.-G.; Wang, X.; Tang, K.; Zeng, L.; et al. Arabidopsis EDM2 Promotes IBM1 Distal Polyadenylation and Regulates Genome DNA Methylation Patterns. *Proc. Natl. Acad. Sci.* **2014**, *111*, 527–532, doi:10.1073/pnas.1320106110.

116. Lee, J.; Yun, J.-Y.; Zhao, W.; Shen, W.-H.; Amasino, R.M. A Methyltransferase Required for Proper Timing of the Vernalization Response in *Arabidopsis*. *Proc. Natl. Acad. Sci.* **2015**, *112*, 2269–2274, doi:10.1073/pnas.1423585112.

117. Kim, Y.J.; Wang, R.; Gao, L.; Li, D.; Xu, C.; Mang, H.; Jeon, J.; Chen, X.; Zhong, X.; Kwak, J.M.; et al. POWERDRESS and HDA9 Interact and Promote Histone H3 Deacetylation at Specific Genomic Sites in *Arabidopsis*. *Proc. Natl. Acad. Sci.* **2016**, *113*, 14858–14863, doi:10.1073/pnas.1618618114.

118. Park, J.; Lim, C.J.; Shen, M.; Park, H.J.; Cha, J.-Y.; Iniesto, E.; Rubio, V.; Mengiste, T.; Zhu, J.-K.; Bressan, R.A.; et al. Epigenetic Switch from Repressive to Permissive Chromatin in Response to Cold Stress. *Proc. Natl. Acad. Sci.* **2018**, *115*, E5400–E5409, doi:10.1073/pnas.1721241115.

119. Ogas, J.; Kaufmann, S.; Henderson, J.; Somerville, C. PICKLE Is a CHD3 Chromatin-Remodeling Factor That Regulates the Transition from Embryonic to Vegetative Development in Arabidopsis. *Proc. Natl. Acad. Sci.* **1999**, *96*, 13839–13844, doi:10.1073/pnas.96.24.13839.

120. Zheng, Y.; Ding, Y.; Sun, X.; Xie, S.; Wang, D.; Liu, X.; Su, L.; Wei, W.; Pan, L.; Zhou, D.-X. Histone Deacetylase HDA9 Negatively Regulates Salt and Drought Stress Responsiveness in Arabidopsis. *J. Exp. Bot.* **2016**, *67*, 1703–1713, doi:10.1093/jxb/erv562.

121. Sarnowski, T.J. AtSWI3B, an Arabidopsis Homolog of SWI3, a Core Subunit of Yeast Swi/Snf Chromatin Remodeling Complex, Interacts with FCA, a Regulator of Flowering Time. *Nucleic Acids Res.* **2002**, *30*, 3412–3421, doi:10.1093/nar/gkf458.

122. Pandey, R.; Müller, A.; Napoli, C.A.; Selinger, D.A.; Pikaard, C.S.; Richards, E.J.; Bender, J.; Mount, D.W.; Jorgensen, R.A. Analysis of Histone Acetyltransferase and Histone Deacetylase Families of Arabidopsis Thaliana Suggests Functional Diversification of Chromatin Modification among Multicellular Eukaryotes. *Nucleic Acids Res.* **2002**, *30*, 5036–5055, doi:10.1093/nar/gkf660.

123. Xie, M.; Ren, G.; Costa-Nunes, P.; Pontes, O.; Yu, B. A Subgroup of SGS3-like Proteins Act Redundantly in RNA-Directed DNA Methylation. *Nucleic Acids Res.* **2012**, *40*, 4422–4431, doi:10.1093/nar/gks034.

124. Nagano, H.; Fukudome, A.; Hiraguri, A.; Moriyama, H.; Fukuhara, T. Distinct Substrate Specificities of Arabidopsis DCL3 and DCL4. *Nucleic Acids Res.* **2014**, *42*, 1845–1856, doi:10.1093/nar/gkt1077.

125. Xu, Y.; Gan, E.-S.; Zhou, J.; Wee, W.-Y.; Zhang, X.; Ito, T. Arabidopsis MRG Domain Proteins Bridge Two Histone Modifications to Elevate Expression of Flowering Genes. *Nucleic Acids Res.* **2014**, *42*, 10960–10974, doi:10.1093/nar/gku781.

126. Li, S.; Liu, L.; Li, S.; Gao, L.; Zhao, Y.; Kim, Y.J.; Chen, X. SUVH1, a Su(Var)3–9 Family Member, Promotes the Expression of Genes Targeted by DNA Methylation. *Nucleic Acids Res.* **2016**, *44*, 608–620, doi:10.1093/nar/gkv958.

127. Lee, W.K.; Cho, M.H. Telomere-Binding Protein Regulates the Chromosome Ends through the Interaction with Histone Deacetylases in *Arabidopsis Thaliana*. *Nucleic Acids Res.* **2016**, *44*, 4610–4624, doi:10.1093/nar/gkw067.

128. Gu, D.; Chen, C.-Y.; Zhao, M.; Zhao, L.; Duan, X.; Duan, J.; Wu, K.; Liu, X. Identification of HDA15-PIF1 as a Key Repression Module Directing the Transcriptional Network of Seed Germination in the Dark. *Nucleic Acids Res.* **2017**, *45*, 7137–7150, doi:10.1093/nar/gkx283.

129. Jin, H.; Choi, S.-M.; Kang, M.-J.; Yun, S.-H.; Kwon, D.-J.; Noh, Y.-S.; Noh, B. Salicylic Acid-Induced Transcriptional Reprogramming by the HAC–NPR1–TGA Histone Acetyltransferase Complex in Arabidopsis. *Nucleic Acids Res.* **2018**, doi:10.1093/nar/gky847.

130. Sako, K.; Kim, J.-M.; Matsui, A.; Nakamura, K.; Tanaka, M.; Kobayashi, M.; Saito, K.; Nishino, N.; Kusano, M.; Taji, T.; et al. Ky-2, a Histone Deacetylase Inhibitor, Enhances High-Salinity Stress Tolerance in *Arabidopsis Thaliana*. *Plant Cell Physiol.* **2016**, *57*, 776–783, doi:10.1093/pcp/pcv199.

131. Sijacic, P.; Holder, D.H.; Bajic, M.; Deal, R.B. *Methyl-CpG-Binding Domain 9 (MBD9) Is Required for H2A.Z Incorporation into Chromatin at a Subset of H2A.Z-Enriched Regions in the Arabidopsis Genome*; Plant Biology, 2018;

132. Springer, N.M.; Napoli, C.A.; Selinger, D.A.; Pandey, R.; Cone, K.C.; Chandler, V.L.; Kaeppler, H.F.; Kaeppler, S.M. Comparative Analysis of SET Domain Proteins in Maize and Arabidopsis Reveals Multiple Duplications Preceding the Divergence of Monocots and Dicots. *Plant Physiol.* **2003**, *132*, 907–925, doi:10.1104/pp.102.013722.

133. Schmitz, R.J.; Tamada, Y.; Doyle, M.R.; Zhang, X.; Amasino, R.M. Histone H2B Deubiquitination Is Required for Transcriptional Activation of *FLOWERING LOCUS C* and for Proper Control of Flowering in Arabidopsis. *Plant Physiol.* **2009**, *149*, 1196–1204, doi:10.1104/pp.108.131508.

134. Airoldi, C.A.; Rovere, F.D.; Falasca, G.; Marino, G.; Kooiker, M.; Altamura, M.M.; Citterio, S.; Kater, M.M. The Arabidopsis BET Bromodomain Factor GTE4 Is Involved in Maintenance of the Mitotic Cell Cycle during Plant Development. *Plant Physiol.* **2010**, *152*, 1320–1334, doi:10.1104/pp.109.150631.

135. Cigliano, R.A.; Cremona, G.; Paparo, R.; Termolino, P.; Perrella, G.; Gutzat, R.; Consiglio, M.F.; Conicella, C. Histone Deacetylase AtHDA7 Is Required for Female Gametophyte and Embryo Development in Arabidopsis. *Plant Physiol.* **2013**, *163*, 431–440, doi:10.1104/pp.113.221713.

136. Sarnowska, E.A.; Rolicka, A.T.; Bucior, E.; Cwiek, P.; Tohge, T.; Fernie, A.R.; Jikumaru, Y.; Kamiya, Y.; Franzen, R.; Schmelzer, E.; et al. DELLA-Interacting SWI3C Core Subunit of Switch/Sucrose Nonfermenting Chromatin Remodeling Complex Modulates Gibberellin Responses and Hormonal Cross Talk in Arabidopsis. *Plant Physiol.* **2013**, *163*, 305–317, doi:10.1104/pp.113.223933.

137. Wang, C.; Dong, X.; Jin, D.; Zhao, Y.; Xie, S.; Li, X.; He, X.; Lang, Z.; Lai, J.; Zhu, J.-K.; et al. Methyl-CpG-Binding Domain Protein MBD7 Is Required for Active DNA Demethylation in Arabidopsis. *Plant Physiol.* **2015**, *167*, 905–914, doi:10.1104/pp.114.252106.

138. Perrella, G.; Carr, C.; Asensi-Fabado, M.A.; Donald, N.A.; Páldi, K.; Hannah, M.A.; Amtmann, A. The Histone Deacetylase Complex 1 Protein of Arabidopsis Has the Capacity to Interact with Multiple Proteins Including Histone 3-Binding Proteins and Histone 1 Variants. *Plant Physiol.* **2016**, *171*, 62–70, doi:10.1104/pp.15.01760.

139. Merini, W.; Romero-Campero, F.J.; Gomez-Zambrano, A.; Zhou, Y.; Turck, F.; Calonje, M. The Arabidopsis Polycomb Repressive Complex 1 (PRC1) Components AtBMI1A, B, and C Impact Gene Networks throughout All Stages of Plant Development. *Plant Physiol.* **2017**, *173*, 627–641, doi:10.1104/pp.16.01259.

140. Kim, D.-H.; Sung, S. The Binding Specificity of the PHD-Finger Domain of VIN3 Moderates Vernalization Response. *Plant Physiol.* **2017**, *173*, 1258–1268, doi:10.1104/pp.16.01320.

141. Chen, L.-Q.; Luo, J.-H.; Cui, Z.-H.; Xue, M.; Wang, L.; Zhang, X.-Y.; Pawlowski, W.P.; He, Y. ATX3, ATX4, and ATX5 Encode Putative H3K4 Methyltransferases and Are Critical for Plant Development. *Plant Physiol.* **2017**, *174*, 1795–1806, doi:10.1104/pp.16.01944.

142. Mayer, K.S.; Chen, X.; Sanders, D.; Chen, J.; Jiang, J.; Nguyen, P.; Scalf, M.; Smith, L.M.; Zhong, X. HDA9-PWR-HOS15 Is a Core Histone Deacetylase Complex Regulating Transcription and Development. *Plant Physiol.* **2019**, *180*, 342–355, doi:10.1104/pp.18.01156.

143. Rocha, P.S.C.F.; Sheikh, M.; Melchiorre, R.; Fagard, M.; Boutet, S.; Loach, R.; Moffatt, B.; Wagner, C.; Vaucheret, H.; Furner, I. The Arabidopsis *HOMOLOGY-DEPENDENT GENE SILENCING1* Gene Codes for an *S* -Adenosyl-l-Homocysteine Hydrolase Required for DNA Methylation-Dependent Gene Silencing. *Plant Cell* **2005**, *17*, 404–417, doi:10.1105/tpc.104.028332.

144. Demidov, D.; Van Damme, D.; Geelen, D.; Blattner, F.R.; Houben, A. Identification and Dynamics of Two Classes of Aurora-Like Kinases in Arabidopsis and Other Plants. *Plant Cell* **2005**, *17*, 836–848, doi:10.1105/tpc.104.029710.

145. Lee, B.; Henderson, D.A.; Zhu, J.-K. The *Arabidopsis* Cold-Responsive Transcriptome and Its Regulation by ICE1. *Plant Cell* **2005**, *17*, 3155–3175, doi:10.1105/tpc.105.035568.

146. Liu, Y.; Koornneef, M.; Soppe, W.J.J. The Absence of Histone H2B Monoubiquitination in the *Arabidopsis Hub1* ( *Rdo4* ) Mutant Reveals a Role for Chromatin Remodeling in Seed Dormancy. *Plant Cell* **2007**, *19*, 433–444, doi:10.1105/tpc.106.049221.

147. Jiang, D.; Yang, W.; He, Y.; Amasino, R.M. *Arabidopsis* Relatives of the Human Lysine-Specific Demethylase1 Repress the Expression of *FWA* and *FLOWERING LOCUS C* and Thus Promote the Floral Transition. *Plant Cell* **2007**, *19*, 2975–2987, doi:10.1105/tpc.107.052373.

148. Li, H.; He, Z.; Lu, G.; Lee, S.C.; Alonso, J.; Ecker, J.R.; Luan, S. A WD40 Domain Cyclophilin Interacts with Histone H3 and Functions in Gene Repression and Organogenesis in *Arabidopsis*. *Plant Cell* **2007**, *19*, 2403–2416, doi:10.1105/tpc.107.053579.

149. Saleh, A.; Alvarez-Venegas, R.; Yilmaz, M.; Le, O.; Hou, G.; Sadder, M.; Al-Abdallat, A.; Xia, Y.; Lu, G.; Ladunga, I.; et al. The Highly Similar *Arabidopsis* Homologs of Trithorax ATX1 and ATX2 Encode Proteins with Divergent Biochemical Functions. *Plant Cell* **2008**, *20*, 568–579, doi:10.1105/tpc.107.056614.

150. Tamada, Y.; Yun, J.-Y.; Woo, S. chul; Amasino, R.M. ARABIDOPSIS TRITHORAX-RELATED7 Is Required for Methylation of Lysine 4 of Histone H3 and for Transcriptional Activation of FLOWERING LOCUS C. *Plant Cell* **2009**, *21*, 3257–3269, doi:10.1105/tpc.109.070060.

151. Liu, C.; Li, L.-C.; Chen, W.-Q.; Chen, X.; Xu, Z.-H.; Bai, S.-N. HDA18 Affects Cell Fate in *Arabidopsis* Root Epidermis via Histone Acetylation at Four Kinase Genes. *Plant Cell* **2013**, *25*, 257–269, doi:10.1105/tpc.112.107045.

152. Wang, Z.; Cao, H.; Sun, Y.; Li, X.; Chen, F.; Carles, A.; Li, Y.; Ding, M.; Zhang, C.; Deng, X.; et al. *Arabidopsis* Paired Amphipathic Helix Proteins SNL1 and SNL2 Redundantly Regulate Primary Seed Dormancy via Abscisic Acid–Ethylene Antagonism Mediated by Histone Deacetylation. *Plant Cell* **2013**, *25*, 149–166, doi:10.1105/tpc.112.108191.

153. López-González, L.; Mouriz, A.; Narro-Diego, L.; Bustos, R.; Martínez-Zapater, J.M.; Jarillo, J.A.; Piñeiro, M. Chromatin-Dependent Repression of the *Arabidopsis* Floral Integrator Genes Involves Plant Specific PHD-Containing Proteins. *Plant Cell* **2014**, *26*, 3922–3938, doi:10.1105/tpc.114.130781.

154. Brosseau, C.; Moffett, P. Functional and Genetic Analysis Identify a Role for Arabidopsis ARGONAUTE5 in Antiviral RNA Silencing. *Plant Cell* **2015**, *27*, 1742–1754, doi:10.1105/tpc.15.00264.

155. Wang, J.; Niu, B.; Huang, J.; Wang, H.; Yang, X.; Dong, A.; Makaroff, C.; Ma, H.; Wang, Y. The PHD Finger Protein MMD1/DUET Ensures the Progression of Male Meiotic Chromosome Condensation and Directly Regulates the Expression of the Condensin Gene *CAP-D3*. *Plant Cell* **2016**, *28*, 1894–1909, doi:10.1105/tpc.16.00040.

156. Hohenstatt, M.L.; Mikulski, P.; Komarynets, O.; Klose, C.; Kycia, I.; Jeltsch, A.; Farrona, S.; Schubert, D. PWWP-DOMAIN INTERACTOR OF POLYCOMBS1 Interacts with Polycomb-Group Proteins and Histones and Regulates Arabidopsis Flowering and Development. *Plant Cell* **2018**, *30*, 117–133, doi:10.1105/tpc.17.00117.

157. Li, H.; Torres-Garcia, J.; Latrasse, D.; Benhamed, M.; Schilderink, S.; Zhou, W.; Kulikova, O.; Hirt, H.; Bisseling, T. Plant-Specific Histone Deacetylases HDT1/2 Regulate *GIBBERELLIN 2-OXIDASE2* Expression to Control Arabidopsis Root Meristem Cell Number. *Plant Cell* **2017**, *29*, 2183–2196, doi:10.1105/tpc.17.00366.

158. Zhang, F.; Wang, L.; Ko, E.E.; Shao, K.; Qiao, H. Histone Deacetylases SRT1 and SRT2 Interact with ENAP1 to Mediate Ethylene-Induced Transcriptional Repression. *Plant Cell* **2018**, *30*, 153–166, doi:10.1105/tpc.17.00671.

159. Mateo-Bonmatí, E.; Esteve-Bruna, D.; Juan-Vicente, L.; Nadi, R.; Candela, H.; Lozano, F.M.; Ponce, M.R.; Pérez-Pérez, J.M.; Micol, J.L. *INCURVATA11* and *CUPULIFORMIS2* Are Redundant Genes That Encode Epigenetic Machinery Components in Arabidopsis. *Plant Cell* **2018**, *30*, 1596–1616, doi:10.1105/tpc.18.00300.

160. Liu, P.; Zhang, S.; Zhou, B.; Luo, X.; Zhou, X.F.; Cai, B.; Jin, Y.H.; Niu, D.; Lin, J.; Cao, X.; et al. The Histone H3K4 Demethylase JMJ16 Represses Leaf Senescence in Arabidopsis. *Plant Cell* **2019**, *31*, 430–443, doi:10.1105/tpc.18.00693.

161. Hernandez-Pinzon, I.; Yelina, N.E.; Schwach, F.; Studholme, D.J.; Baulcombe, D.; Dalmay, T. SDE5, the Putative Homologue of a Human MRNA Export Factor, Is Required for Transgene Silencing and Accumulation of Trans-Acting Endogenous SiRNA: SDE5 Is Involved in PTGS and TasiRNA Biogenesis. *Plant J.* **2007**, *50*, 140–148, doi:10.1111/j.1365-313X.2007.03043.x.

162. Earley, K.W.; Shook, M.S.; Brower‐Toland, B.; Hicks, L.; Pikaard, C.S. In Vitro Specificities of Arabidopsis Co-Activator Histone Acetyltransferases: Implications for Histone Hyperacetylation in Gene Activation. *Plant J.* **2007**, *52*, 615–626, doi:10.1111/j.1365-313X.2007.03264.x.

163. Gu, X.; Jiang, D.; Wang, Y.; Bachmair, A.; He, Y. Repression of the Floral Transition via Histone H2B Monoubiquitination. *Plant J.* **2009**, *57*, 522–533, doi:10.1111/j.1365-313X.2008.03709.x.

164. Tran, H.T.; Nimick, M.; Uhrig, R.G.; Templeton, G.; Morrice, N.; Gourlay, R.; DeLong, A.; Moorhead, G.B.G. Arabidopsis Thaliana Histone Deacetylase 14 (HDA14) Is an α-Tubulin Deacetylase That Associates with PP2A and Enriches in the Microtubule Fraction with the Putative Histone Acetyltransferase ELP3: Arabidopsis PP2A, HDA14 and ELP3 Bind Microtubules. *Plant J.* **2012**, *71*, 263–272, doi:10.1111/j.1365-313X.2012.04984.x.

165. Zhao, Q.-Q.; Lin, R.-N.; Li, L.; Chen, S.; He, X.-J. A Methylated-DNA-Binding Complex Required for Plant Development Mediates Transcriptional Activation of Promoter Methylated Genes. *J. Integr. Plant Biol.* **2019**, *61*, 120–139, doi:10.1111/jipb.12767.

166. Xiao, X.; Zhang, J.; Li, T.; Fu, X.; Satheesh, V.; Niu, Q.; Lang, Z.; Zhu, J.-K.; Lei, M. A Group of SUVH Methyl-DNA Binding Proteins Regulate Expression of the DNA Demethylase ROS1 in *Arabidopsis*: SUVHs Regulate Active DNA Demethylation. *J. Integr. Plant Biol.* **2019**, *61*, 110–119, doi:10.1111/jipb.12768.

167. Kang, H.; Zhang, C.; An, Z.; Shen, W.; Zhu, Y. AtINO80 and AtARP5 Physically Interact and Play Common as Well as Distinct Roles in Regulating Plant Growth and Development. *New Phytol.* **2019**, *223*, 336–353, doi:10.1111/nph.15780.

168. Luo, M.; Tai, R.; Yu, C.; Yang, S.; Chen, C.; Lin, W.; Schmidt, W.; Wu, K. Regulation of Flowering Time by the Histone Deacetylase HDA 5 in A Rabidopsis. *Plant J.* **2015**, *82*, 925–936, doi:10.1111/tpj.12868.

169. Deng, S.; Xu, J.; Liu, J.; Kim, S.-H.; Shi, S.; Chua, N.-H. JMJ24 Binds to RDR2 and Is Required for the Basal Level Transcription of Silenced Loci in Arabidopsis. *Plant J.* **2015**, *83*, 770–782, doi:10.1111/tpj.12924.

170. Waterworth, W.M.; Drury, G.E.; Blundell-Hunter, G.; West, C.E. Arabidopsis TAF1 Is an MRE11-Interacting Protein Required for Resistance to Genotoxic Stress and Viability of the Male Gametophyte. *Plant J.* **2015**, *84*, 545–557, doi:10.1111/tpj.13020.

171. Dutta, A.; Choudhary, P.; Caruana, J.; Raina, R. JMJ27, an Arabidopsis H3K9 Histone Demethylase, Modulates Defense against *Pseudomonas Syringae* and Flowering Time. *Plant J.* **2017**, *91*, 1015–1028, doi:10.1111/tpj.13623.

172. Latrasse, D.; Benhamed, M.; Henry, Y.; Domenichini, S.; Kim, W.; Zhou, D.-X.; Delarue, M. The MYST Histone Acetyltransferases Are Essential for Gametophyte Development in Arabidopsis. *BMC Plant Biol.* **2008**, *8*, 121, doi:10.1186/1471-2229-8-121.

173. Bieluszewski, T.; Galganski, L.; Sura, W.; Bieluszewska, A.; Abram, M.; Ludwikow, A.; Ziolkowski, P.; Sadowski, J. AtEAF1 Is a Potential Platform Protein for Arabidopsis NuA4 Acetyltransferase Complex. *BMC Plant Biol.* **2015**, *15*, 75, doi:10.1186/s12870-015-0461-1.

174. Veiseth, S.V.; Rahman, M.A.; Yap, K.L.; Fischer, A.; Egge-Jacobsen, W.; Reuter, G.; Zhou, M.-M.; Aalen, R.B.; Thorstensen, T. The SUVR4 Histone Lysine Methyltransferase Binds Ubiquitin and Converts H3K9me1 to H3K9me3 on Transposon Chromatin in Arabidopsis. *PLOS Genet.* **2011**, *7*, e1001325, doi:10.1371/journal.pgen.1001325.

175. Nishimura, T.; Molinard, G.; Petty, T.J.; Broger, L.; Gabus, C.; Halazonetis, T.D.; Thore, S.; Paszkowski, J. Structural Basis of Transcriptional Gene Silencing Mediated by Arabidopsis MOM1. *PLoS Genet.* **2012**, *8*, e1002484, doi:10.1371/journal.pgen.1002484.

176. Yang, H.; Han, Z.; Cao, Y.; Fan, D.; Li, H.; Mo, H.; Feng, Y.; Liu, L.; Wang, Z.; Yue, Y.; et al. A Companion Cell–Dominant and Developmentally Regulated H3K4 Demethylase Controls Flowering Time in Arabidopsis via the Repression of FLC Expression. *PLOS Genet.* **2012**, *8*, e1002664, doi:10.1371/journal.pgen.1002664.

177. Caro, E.; Stroud, H.; Greenberg, M.V.C.; Bernatavichute, Y.V.; Feng, S.; Groth, M.; Vashisht, A.A.; Wohlschlegel, J.; Jacobsen, S.E. The SET-Domain Protein SUVR5 Mediates H3K9me2 Deposition and Silencing at Stimulus Response Genes in a DNA Methylation–Independent Manner. *PLoS Genet.* **2012**, *8*, e1002995, doi:10.1371/journal.pgen.1002995.

178. Molitor, A.M.; Bu, Z.; Yu, Y.; Shen, W.-H. Arabidopsis AL PHD-PRC1 Complexes Promote Seed Germination through H3K4me3-to-H3K27me3 Chromatin State Switch in Repression of Seed Developmental Genes. *PLOS Genet.* **2014**, *10*, e1004091, doi:10.1371/journal.pgen.1004091.

179. Zheng, B.; He, H.; Zheng, Y.; Wu, W.; McCormick, S. An ARID Domain-Containing Protein within Nuclear Bodies Is Required for Sperm Cell Formation in Arabidopsis Thaliana. *PLoS Genet.* **2014**, *10*, e1004421, doi:10.1371/journal.pgen.1004421.

180. Liang, S.C.; Hartwig, B.; Perera, P.; Mora-García, S.; de Leau, E.; Thornton, H.; de Alves, F.L.; Rapsilber, J.; Yang, S.; James, G.V.; et al. Kicking against the PRCs – A Domesticated Transposase Antagonises Silencing Mediated by Polycomb Group Proteins and Is an Accessory Component of Polycomb Repressive Complex 2. *PLOS Genet.* **2015**, *11*, e1005660, doi:10.1371/journal.pgen.1005660.

181. Liu, Z.-W.; Zhou, J.-X.; Huang, H.-W.; Li, Y.-Q.; Shao, C.-R.; Li, L.; Cai, T.; Chen, S.; He, X.-J. Two Components of the RNA-Directed DNA Methylation Pathway Associate with MORC6 and Silence Loci Targeted by MORC6 in Arabidopsis. *PLOS Genet.* **2016**, *12*, e1006026, doi:10.1371/journal.pgen.1006026.

182. Liao, Y.-W.-K.; Sun, Z.-H.; Zhou, Y.-H.; Shi, K.; Li, X.; Zhang, G.-Q.; Xia, X.-J.; Chen, Z.-X.; Yu, J.-Q. The Role of Hydrogen Peroxide and Nitric Oxide in the Induction of Plant-Encoded RNA-Dependent RNA Polymerase 1 in the Basal Defense against Tobacco Mosaic Virus. *PLoS ONE* **2013**, *8*, e76090, doi:10.1371/journal.pone.0076090.

183. Li, J.; Wang, Z.; Hu, Y.; Cao, Y.; Ma, L. Polycomb Group Proteins RING1A and RING1B Regulate the Vegetative Phase Transition in Arabidopsis. *Front. Plant Sci.* **2017**, *8*, doi:10.3389/fpls.2017.00867.

184. Fina, J.P.; Masotti, F.; Rius, S.P.; Crevacuore, F.; Casati, P. HAC1 and HAF1 Histone Acetyltransferases Have Different Roles in UV-B Responses in Arabidopsis. *Front. Plant Sci.* **2017**, *8*, doi:10.3389/fpls.2017.01179.

185. Henikoff, S.; Comai, L. A DNA Methyltransferase Homolog with a Chromodomain Exists in Multiple Polymorphic Forms in Arabidopsis. *Genetics* **1998**, *149*, 307–318.

186. Duque, P.; Chua, N.-H. IMB1, a Bromodomain Protein Induced during Seed Imbibition, Regulates ABA- and PhyA-Mediated Responses of Germination in *Arabidopsis*. *Plant J.* **2003**, *35*, 787–799, doi:10.1046/j.1365-313X.2003.01848.x.
