# Supplementary material for: A Systems Biology Approach to Identify Essential Epigenetic Regulators for Specific Biological Processes in Plants
Source: Plants (Basel). 2021 Feb 13;10(2):364. doi: 10.3390/plants10020364 (PMC7918732; doi:10.3390/plants10020364)
Supplement: Supplementary file 1 [file plants-10-00364-s001.zip › SupplementalResults_Final.docx]

**SUPPLEMENTAL RESULTS**

**Description of epigenetic regulators**

***DNA methylation and demethylation***

DNA methylation, methylation of cytosine in genomic DNA, is an epigenetic mark important for stability of genomes and repression of gene expression [1]. In plants, DNA methylation is added by *de novo* or maintenance DNA methylation pathways and removed through active DNA demethylation pathways. *De novo* DNA methylation is established by the RNA-directed DNA methylation (RdDM) pathway, in which long noncoding RNAs and siRNAs mediate sequence-specific cytosine methylation [2]. RdDM is best characterized in Arabidopsis, where it targets heterochromatic regions that are enriched in transposable elements (TEs) and other DNA repeat sequences [3]. Maintenance of methylation marks depends on the cytosine context, wherein CG and CHG (H stands for A, T, C) methylation is maintained by DNA METHYLTRANSFERASE 1 (MET1) and CHROMOMETHYLASE 3 (CMT3), respectively, while CHH methylation is catalyzed by CMT2 or established *de novo* by DOMAINS REARRANGED METHYLTRANSFERASE 2 (DRM2) in every cell cycle [4,5]. To remove methylation, plants utilize a multistep base excision repair pathway initiated by a family of 5-methylcytosine DNA glycosylases including ROS1 [6,7].

***Histone modification***

Histone variants and posttranslational modification of histone N-terminal tails are known to affect chromatin organization and control gene expression. It was proposed that the combination of histone modifications at a given genomic locus forms the ‘histone code’ to extend the information potential of DNA [8] and affect gene regulation, which is an active research area with applications in health, food security, and environment. Well-characterized histone modifications in plants include histone acetylation, methylation and ubiquitination.

***Histone acetylation:*** Histone lysine (K) acetylation reduces the positive charge of histones, weakening its interaction with DNA. Loose DNA-histone interaction leads to ‘open chromatin’, which is generally associated with active genes and transcription. Histone acetylation is added by histone acetyltransferases (HATs) and erased by histone deacetylases (HDAC) [9]. Plants have three families of HDACs: HDA1/RPD3, SIR2 and the plant specific HD2/HDT [9]. Among these, the HDA1/RPD3 family HDACs are the best-characterized. For example, HDA6 is involved in flowering control [10], transcriptional gene silencing, rRNA gene repression, nucleolar dominance [11], and stress response [12]. Likewise, the HDA1/RPD3 homologs HDA9 and HDA19 regulate stress response, flowering [13], development, and the circadian clock [14,15]. Recent studies of the plant-specific HD2 histone deacetylases show diverse roles in plants. For example, HDT1/HD2A and HDT2/HD2B regulate root development [16]; HDT2 is involved in plant innate immunity [17]; HDT2 and HDT3 modulate rRNA processing at both transcriptional and posttranscriptional level; and HDT4 (with HDA6) regulate telomeric length through interaction with telomere-binding proteins [19]. Finally, the pair of plant homologs of sirtuin-type lysine deacetylases (SRT1 and SRT2) are involved in ethylene-induced transcriptional repression [20] while SRT2 was found to have roles in mitochondrial energy metabolism [21].

***Histone methylation:*** Methylation of histone tails is a posttranslational modification associated with gene activation, gene repression, heterochromatin maintenance, and control of transposon activity. Histone methylation is reversible due to the function of highly conserved methyltransferases and demethylases, using both arginine (R) and lysine (K) residues as substrate. Plants possess a superfamily of histone lysine methyltransferases which share the SET (**S**u(var)3-9, **E**nhancer of Zeste, **T**rithorax) domain. These enzymes catalyze SAM-dependent addition of methyl group to lysine, which can be mono-, di- or trimethylated, in lysine residues at histone tails. In plants, the best studied lysine residues are in histone H3 at K4, K9, K27 and K36. Conserved families of SET domain-containing proteins (SDG) are responsible for depositing histone methylation marks and have been reviewed extensively [22–24].

H3K4 methylation, a mark associated with active gene expression, is deposited by trithorax group (TRX) subgroup of SDG proteins such as ARABIDOPSIS TRITHORAX RELATED 7 (ATXR7/SDG25) for histone H3 lysine 4 monomethylation (H3K4me1,[25]), ATX2 for H3K4me2 [26][24]; and ATX1, SDG2, SDG4, ATXR7/SDG25, and SDG26 for H3K4me3 [24]. Another methylation mark associated with active gene transcription is H3K36 methylation. The ABSENT, SMALL, HOMEOTIC 1 (ASH1)-related homolog ASHR3/SDG4 is an H3K36 methyltransferase that deposits mono-, di- and try-methylation [27,28] while the ASH1 homolog ASHH2/SDG8 catalyzes di- and tri-methylation of H3K36 within gene bodies [29,30].

H3K27me3 is a repressive histone mark deposited by the Polycomb Repressive Complex 2 (PRC2). Plant homologs of Drosophila’s Enhancer of Zeste (E(Z)) - CURLY LEAF (CLF), SWINGER (SWN) or MEDEA (MEA) - are responsible for the catalytic activity of PRC2 complex [31–33]. ATXR5 and ATXR6, on the other hand, are writers of H3K27me1 on histone H3.1 [34], a mark that was shown to associate with the replication fork and maintain the memory of silencing during DNA replication [35].

Methylation of H3K9 is mediated by the largest subgroup of SDG proteins, Su(var)3-9-related proteins, in Arabidopsis. KRYPTONITE/SUVH4 deposits H3K9me1 and H3K9me2, binds methylated cytosines, and cooperates with the CHG DNA methyltransferase CMT3 to maintain both repressive marks in silenced loci [36]. Similarly, SUVH5 and SUVH6 [37] were shown to contribute to H3K9me1 and H3K9me2 at different heterochromatic sequences. SUVR4 was a reported writer of H3K9me3 [38] involved in silencing transposons and pseudogenes.

Arginine residue of the histone tail is another methylation site associated with gene regulation in plants. This modification exists as monomethyl-arginine and dimethyl-arginine which can be asymmetric or symmetric [39]. These reactions are catalyzed by protein arginine methyltransferases (PRMTs). Arabidopsis has 11 PRMTs, including the plant-specific PRMT10 [40]. Among these, *in vitro* histone methyltransferase activity was reported for PRMT1a, PRMT1b [41] and PRMT10 [42], all of which can methylate H4R3. In addition, *in vivo* activity of PRMTs were shown through work on PRMT4a and PRMT4b, which redundantly affect asymmetrically di-methylated H3R17 and H3R26 and control flowering time [42]. Moreover, PRMT5 can symmetrically di-methylate histone H4R3 *in vivo*, with roles in vernalization [43] and iron homeostasis [44].

Removal of histone methylation is achieved by two groups of proteins: lysine-specific histone demethylase-LIKE (LDL) proteins and the JUMONJI-C-DOMAIN (JmjC) proteins. The Arabidopsis genome encodes 4 LDL histone demethylases and 21 JmjC proteins [45]. The LDL proteins FLOWERING LOCUS D (FLD) and its close homologs LDL1 and LDL2 promote floral transition through its involvement with demethylation of H3K4 in *FLOWERING LOCUS C* (FLC) [46]. LDL3 was shown to specifically eliminate H3K4me2 during callus formation in roots, thereby priming and facilitating the activation of genes during shoot induction [47]. On the other hand, RELATIVE OF EARLY FLOWERING 6 (REF6) and its close paralogs ELF6 and JMJ13, are Jumonji-type histone demethylases that determine the boundaries established by PRC2-mediated gene repression by removing and limiting the spreading of H3K27me3 [48–50]. Other characterized JmjC proteins include the H3K4 demethylases JMJ14 [51] and JMJ18 [52] that promote flowering; H3K4 demethylases JMJ16 and JMJ17 involved in leaf senescence [53] and drought [54], respectively; and INCREASE IN BONSAI METHYLATION (IBM1), a histone H3K9 demethylase involved in DNA methylation [55].

***Histone ubiquitination:*** Histone H2A and H2B are substrates for histone mono-ubiquitination. In Arabidopsis, HISTONE MONOUBIQUITINATION 1 (AtHUB1) and AtHUB2 are RING E3 ligase homologs that function in the mono-ubiquitination of histone H2B and regulates a variety of processes . Histone ubiquitination is reversible through the action of histone deubiquitinases. Indeed, several deubiquitinases are known to target H2B in Arabidopsis: ubiquitin-specific protease 22 (UBP22)[59], UBP26[60] and OTUBAIN-LIKE DEUBIQUITINASE (OTLD1)[61].

***Chromatin/nucleosome remodeling***

Chromatin remodelers move, eject, or restructure the configuration of nucleosomes, using ATP as the energy source [62]. In eukaryotes, there are four families of ATPase-driven chromatin remodelers: SWI/SNF, ISWI, CHD and INO80/SWR1.

SWItch/Sucrose Non Fermentable (SWI/SNF) chromatin remodelers slide and eject nucleosomes at many loci [62]. SWI/SNF in plants come in three forms: BRAHMA (BRM), SPLAYED (SYD) and MINUSCULE (MINU) [63]. BRM functions as a regulator of Polycomb function during developmental and reproductive processes [64–66]. SYD is involved in regulating shoot apical meristem identity [67], biotic stress signaling [68] and like BRM, can overcome Polycomb repression to co-establish floral organ identity [69]. Arabidopsis MINU1 and MINU2 are involved in temporary growth arrest upon perceiving stress [70] and double mutants cause embryogenesis and stem cell maintenance defects [71].

Two ISWI homologs are present in Arabidopsis: CHROMATIN REMODELING PROTEIN 11 (CHR11) and CHR17. The ISWI proteins have a conserved role of sliding nucleosomes in gene bodies to form patterns [72] and replacing histone variants H2A.Z in gene bodies [73].

The CHD1 (chromodomain, helicase/ATPase and DNA‐binding domain) family homologs in Arabidopsis are CHD1/CHR5 and three CHD3 types: PICKLE (PKL), PICKLE RELATED 1 (PKR1) and PKR2 [63]. CHD1 is involved in reducing nucleosome occupancy at transcriptional start sites and was shown to directly activate the expression of seed maturation genes [74,75]. PKL is an ATPase chromatin remodeler that interacts with PHOTOPERIOD INDEPENDENT EARLY FLOWERING 1 (PIE1) to promote retention of the repressive mark H3K27me3 in H2A.Z-targeted loci [76], and regulates many developmental processes [77–79] and stress response [80,81].

Lastly, plants possess chromatin remodeling complexes that process H2A.Z, including the SWI/SNF2-RELATED 1 (SWR1) which deposits H2A.Z [82] and INO80 (INOSITOL REQUIRING 80) complex which evicts H2A.Z [83].

***Proteins involved in siRNA biogenesis***

Non-coding RNA that participate in chromatin regulation include long non-coding RNAs (lncRNAs; > 200 nt) and small RNAs (sRNAs; 18-30 nt). Some lncRNAs mediate chromatin modifications, for example, COLDAIR, an intronic lncRNA in the sense strand of FLOWERING LOCUS C (FLC), transiently recruits the PRC2 complex to FLC during cold-mediated epigenetic repression of FLC [84]. The sRNAs classify into two broad classes: miRNAs and siRNAs. siRNAs specifically have a role in transcriptional gene silencing with relevance to epigenetic silencing. In canonical RNA-directed DNA methylation pathway (RdDM) pathway [3], POL IV generates transcripts which serve as template for RNA-DEPENDENT RNA POLYMERASE 1 (RDR1) to synthesize double-stranded RNAs. DICER-LIKE3 (DCL3) cleaves the double-stranded RNA to form 24-nt siRNAs that are loaded to ARGONAUTE (AGO) proteins, mainly AGO4 and AGO6. The siRNA-AGO complex then binds to target loci through base-pairing with POL V-synthesized lncRNAs (scaffolds). The RNA scaffold complex recruits other protein complex including DRM2, the DNA methyltransferase responsible for *de novo* DNA methylation [3] and leads to subsequent transcriptional gene silencing.

**REFERENCE**

1. Zhang, H.; Lang, Z.; Zhu, J.-K. Dynamics and Function of DNA Methylation in Plants. *Nat. Rev. Mol. Cell Biol.* **2018**, *19*, 489–506, doi:10.1038/s41580-018-0016-z.

2. Zhang, H.; Zhu, J.-K. RNA-Directed DNA Methylation. *Curr. Opin. Plant Biol.* **2011**, *14*, 142–147, doi:10.1016/j.pbi.2011.02.003.

3. Matzke, M.A.; Mosher, R.A. RNA-Directed DNA Methylation: An Epigenetic Pathway of Increasing Complexity. *Nat. Rev. Genet.* **2014**, *15*, 394–408, doi:10.1038/nrg3683.

4. Zemach, A.; Kim, M.Y.; Hsieh, P.-H.; Coleman-Derr, D.; Eshed-Williams, L.; Thao, K.; Harmer, S.L.; Zilberman, D. The Arabidopsis Nucleosome Remodeler DDM1 Allows DNA Methyltransferases to Access H1-Containing Heterochromatin. *Cell* **2013**, *153*, 193–205, doi:10.1016/j.cell.2013.02.033.

5. Law, J.A.; Jacobsen, S.E. Establishing, Maintaining and Modifying DNA Methylation Patterns in Plants and Animals. *Nat. Rev. Genet.* **2010**, *11*, 204–220, doi:10.1038/nrg2719.

6. Gong, Z.; Morales-Ruiz, T.; Ariza, R.R.; Roldán-Arjona, T.; David, L.; Zhu, J.-K. ROS1, a Repressor of Transcriptional Gene Silencing in Arabidopsis, Encodes a DNA Glycosylase/Lyase. *Cell* **2002**, *111*, 803–814, doi:10.1016/S0092-8674(02)01133-9.

7. Gehring, M.; Huh, J.H.; Hsieh, T.-F.; Penterman, J.; Choi, Y.; Harada, J.J.; Goldberg, R.B.; Fischer, R.L. DEMETER DNA Glycosylase Establishes MEDEA Polycomb Gene Self-Imprinting by Allele-Specific Demethylation. *Cell* **2006**, *124*, 495–506, doi:10.1016/j.cell.2005.12.034.

8. Jenuwein, T.; Allis, C.D. Translating the Histone Code. *Science* **2001**, *293*, 1074–1080, doi:10.1126/science.1063127.

9. Pandey, R.; Müller, A.; Napoli, C.A.; Selinger, D.A.; Pikaard, C.S.; Richards, E.J.; Bender, J.; Mount, D.W.; Jorgensen, R.A. Analysis of Histone Acetyltransferase and Histone Deacetylase Families of Arabidopsis Thaliana Suggests Functional Diversification of Chromatin Modification among Multicellular Eukaryotes. *Nucleic Acids Res.* **2002**, *30*, 5036–5055.

10. He, Y.; Michaels, S.D.; Amasino, R.M. Regulation of Flowering Time by Histone Acetylation in Arabidopsis. *Science* **2003**, *302*, 1751–1754, doi:10.1126/science.1091109.

11. Pikaard, C.S.; Scheid, O.M. Epigenetic Regulation in Plants. *Cold Spring Harb. Perspect. Biol.* **2014**, *6*, a019315, doi:10.1101/cshperspect.a019315.

12. Chen, L.-T.; Luo, M.; Wang, Y.-Y.; Wu, K. Involvement of Arabidopsis Histone Deacetylase HDA6 in ABA and Salt Stress Response. *J. Exp. Bot.* **2010**, *61*, 3345–3353, doi:10.1093/jxb/erq154.

13. Zheng, Y.; Ding, Y.; Sun, X.; Xie, S.; Wang, D.; Liu, X.; Su, L.; Wei, W.; Pan, L.; Zhou, D.-X. Histone Deacetylase HDA9 Negatively Regulates Salt and Drought Stress Responsiveness in Arabidopsis. *J. Exp. Bot.* **2016**, *67*, 1703–1713, doi:10.1093/jxb/erv562.

14. Hu, Y.; Lu, Y.; Zhao, Y.; Zhou, D.-X. Histone Acetylation Dynamics Integrates Metabolic Activity to Regulate Plant Response to Stress. *Front. Plant Sci.* **2019**, *10*, doi:10.3389/fpls.2019.01236.

15. Long, J.A.; Ohno, C.; Smith, Z.R.; Meyerowitz, E.M. TOPLESS Regulates Apical Embryonic Fate in Arabidopsis. *Science* **2006**, *312*, 1520–1523, doi:10.1126/science.1123841.

16. Li, H.; Torres-Garcia, J.; Latrasse, D.; Benhamed, M.; Schilderink, S.; Zhou, W.; Kulikova, O.; Hirt, H.; Bisseling, T. Plant-Specific Histone Deacetylases HDT1/2 Regulate GIBBERELLIN 2-OXIDASE2 Expression to Control Arabidopsis Root Meristem Cell Number. *Plant Cell* **2017**, *29*, 2183–2196, doi:10.1105/tpc.17.00366.

17. Latrasse, D.; Jégu, T.; Li, H.; de Zelicourt, A.; Raynaud, C.; Legras, S.; Gust, A.; Samajova, O.; Veluchamy, A.; Rayapuram, N.; et al. MAPK-Triggered Chromatin Reprogramming by Histone Deacetylase in Plant Innate Immunity. *Genome Biol.* **2017**, *18*, 131, doi:10.1186/s13059-017-1261-8.

18. Chen, X.; Lu, L.; Qian, S.; Scalf, M.; Smith, L.M.; Zhong, X. Canonical and Noncanonical Actions of Arabidopsis Histone Deacetylases in Ribosomal RNA Processing. *Plant Cell* **2018**, *30*, 134–152, doi:10.1105/tpc.17.00626.

19. Lee, W.K.; Cho, M.H. Telomere-Binding Protein Regulates the Chromosome Ends through the Interaction with Histone Deacetylases in Arabidopsis Thaliana. *Nucleic Acids Res.* **2016**, *44*, 4610–4624, doi:10.1093/nar/gkw067.

20. Zhang, F.; Wang, L.; Ko, E.E.; Shao, K.; Qiao, H. Histone Deacetylases SRT1 and SRT2 Interact with ENAP1 to Mediate Ethylene-Induced Transcriptional Repression. *Plant Cell* **2018**, *30*, 153–166, doi:10.1105/tpc.17.00671.

21. König, A.-C.; Hartl, M.; Pham, P.A.; Laxa, M.; Boersema, P.J.; Orwat, A.; Kalitventseva, I.; Plöchinger, M.; Braun, H.-P.; Leister, D.; et al. The Arabidopsis Class II Sirtuin Is a Lysine Deacetylase and Interacts with Mitochondrial Energy Metabolism. *Plant Physiol.* **2014**, *164*, 1401–1414, doi:10.1104/pp.113.232496.

22. Pontvianne, F.; Blevins, T.; Pikaard, C.S. Arabidopsis Histone Lysine Methyltransferases. In *Advances in Botanical Research*; Kader, J.-C., Delseny, M., Eds.; Advances in Botanical Research; Academic Press, 2010; Vol. 53, pp. 1–22.

23. Liu, C.; Lu, F.; Cui, X.; Cao, X. Histone Methylation in Higher Plants. *Annu. Rev. Plant Biol.* **2010**, *61*, 395–420, doi:10.1146/annurev.arplant.043008.091939.

24. Xiao, J.; Lee, U.-S.; Wagner, D. Tug of War: Adding and Removing Histone Lysine Methylation in Arabidopsis. *Curr. Opin. Plant Biol.* **2016**, *34*, 41–53, doi:10.1016/j.pbi.2016.08.002.

25. Tamada, Y.; Yun, J.-Y.; Woo, S. chul; Amasino, R.M. ARABIDOPSIS TRITHORAX-RELATED7 Is Required for Methylation of Lysine 4 of Histone H3 and for Transcriptional Activation of FLOWERING LOCUS C. *Plant Cell* **2009**, *21*, 3257–3269, doi:10.1105/tpc.109.070060.

26. Saleh, A.; Alvarez-Venegas, R.; Yilmaz, M.; Le, O.; Hou, G.; Sadder, M.; Al-Abdallat, A.; Xia, Y.; Lu, G.; Ladunga, I.; et al. The Highly Similar Arabidopsis Homologs of Trithorax ATX1 and ATX2 Encode Proteins with Divergent Biochemical Functions. *Plant Cell* **2008**, *20*, 568–579, doi:10.1105/tpc.107.056614.

27. Kumpf, R.; Thorstensen, T.; Rahman, M.A.; Heyman, J.; Nenseth, H.Z.; Lammens, T.; Herrmann, U.; Swarup, R.; Veiseth, S.V.; Emberland, G.; et al. The ASH1-RELATED3 SET-Domain Protein Controls Cell Division Competence of the Meristem and the Quiescent Center of the Arabidopsis Primary Root. *Plant Physiol.* **2014**, *166*, 632–643, doi:10.1104/pp.114.244798.

28. Cartagena, J.A.; Matsunaga, S.; Seki, M.; Kurihara, D.; Yokoyama, M.; Shinozaki, K.; Fujimoto, S.; Azumi, Y.; Uchiyama, S.; Fukui, K. The Arabidopsis SDG4 Contributes to the Regulation of Pollen Tube Growth by Methylation of Histone H3 Lysines 4 and 36 in Mature Pollen. *Dev. Biol.* **2008**, *315*, 355–368, doi:10.1016/j.ydbio.2007.12.016.

29. Li, Y.; Mukherjee, I.; Thum, K.E.; Tanurdzic, M.; Katari, M.S.; Obertello, M.; Edwards, M.B.; McCombie, W.R.; Martienssen, R.A.; Coruzzi, G.M. The Histone Methyltransferase SDG8 Mediates the Epigenetic Modification of Light and Carbon Responsive Genes in Plants. *Genome Biol.* **2015**, *16*, 79, doi:10.1186/s13059-015-0640-2.

30. Xu, L.; Zhao, Z.; Dong, A.; Soubigou-Taconnat, L.; Renou, J.-P.; Steinmetz, A.; Shen, W.-H. Di- and Tri- but Not Monomethylation on Histone H3 Lysine 36 Marks Active Transcription of Genes Involved in Flowering Time Regulation and Other Processes in Arabidopsis Thaliana. *Mol. Cell. Biol.* **2008**, *28*, 1348–1360, doi:10.1128/MCB.01607-07.

31. Chanvivattana, Y.; Bishopp, A.; Schubert, D.; Stock, C.; Moon, Y.-H.; Sung, Z.R.; Goodrich, J. Interaction of Polycomb-Group Proteins Controlling Flowering in Arabidopsis. *Development* **2004**, *131*, 5263–5276, doi:10.1242/dev.01400.

32. Grossniklaus, U. Maternal Control of Embryogenesis by MEDEA, a Polycomb Group Gene in Arabidopsis. *Science* **1998**, *280*, 446–450, doi:10.1126/science.280.5362.446.

33. Makarevich, G.; Leroy, O.; Akinci, U.; Schubert, D.; Clarenz, O.; Goodrich, J.; Grossniklaus, U.; Köhler, C. Different Polycomb Group Complexes Regulate Common Target Genes in Arabidopsis. *EMBO Rep.* **2006**, *7*, 947–952, doi:10.1038/sj.embor.7400760.

34. Jacob, Y.; Feng, S.; LeBlanc, C.A.; Bernatavichute, Y.V.; Stroud, H.; Cokus, S.; Johnson, L.M.; Pellegrini, M.; Jacobsen, S.E.; Michaels, S.D. ATXR5 and ATXR6 Are H3K27 Monomethyltransferases Required for Chromatin Structure and Gene Silencing. *Nat. Struct. Mol. Biol.* **2009**, *16*, 763–768, doi:10.1038/nsmb.1611.

35. Jiang, D.; Berger, F. DNA Replication–Coupled Histone Modification Maintains Polycomb Gene Silencing in Plants. *Science* **2017**, *357*, 1146–1149, doi:10.1126/science.aan4965.

36. Du, J.; Johnson, L.M.; Groth, M.; Feng, S.; Hale, C.J.; Li, S.; Vashisht, A.A.; Gallego-Bartolome, J.; Wohlschlegel, J.A.; Patel, D.J.; et al. Mechanism of DNA Methylation-Directed Histone Methylation by KRYPTONITE. *Mol. Cell* **2014**, *55*, 495–504, doi:10.1016/j.molcel.2014.06.009.

37. Ebbs, M.L.; Bender, J. Locus-Specific Control of DNA Methylation by the Arabidopsis SUVH5 Histone Methyltransferase. *Plant Cell* **2006**, *18*, 1166–1176, doi:10.1105/tpc.106.041400.

38. Veiseth, S.V.; Rahman, M.A.; Yap, K.L.; Fischer, A.; Egge-Jacobsen, W.; Reuter, G.; Zhou, M.-M.; Aalen, R.B.; Thorstensen, T. The SUVR4 Histone Lysine Methyltransferase Binds Ubiquitin and Converts H3K9me1 to H3K9me3 on Transposon Chromatin in Arabidopsis. *PLOS Genet.* **2011**, *7*, e1001325, doi:10.1371/journal.pgen.1001325.

39. Bedford, M.T.; Richard, S. Arginine Methylation: An Emerging Regulatorof Protein Function. *Mol. Cell* **2005**, *18*, 263–272, doi:10.1016/j.molcel.2005.04.003.

40. Ahmad, A.; Cao, X. Plant PRMTs Broaden the Scope of Arginine Methylation. *J. Genet. Genomics* **2012**, *39*, 195–208, doi:10.1016/j.jgg.2012.04.001.

41. Yan, D.; Zhang, Y.; Niu, L.; Yuan, Y.; Cao, X. Identification and Characterization of Two Closely Related Histone H4 Arginine 3 Methyltransferases in Arabidopsis Thaliana. *Biochem. J.* **2007**, *408*, 113–121, doi:10.1042/BJ20070786.

42. Niu, L.; Zhang, Y.; Pei, Y.; Liu, C.; Cao, X. Redundant Requirement for a Pair of PROTEIN ARGININE METHYLTRANSFERASE4 Homologs for the Proper Regulation of Arabidopsis Flowering Time. *Plant Physiol.* **2008**, *148*, 490–503, doi:10.1104/pp.108.124727.

43. Schmitz, R.J.; Sung, S.; Amasino, R.M. Histone Arginine Methylation Is Required for Vernalization-Induced Epigenetic Silencing of FLC in Winter-Annual Arabidopsis Thaliana. *Proc. Natl. Acad. Sci.* **2008**, *105*, 411–416, doi:10.1073/pnas.0710423104.

44. Fan, H.; Zhang, Z.; Wang, N.; Cui, Y.; Sun, H.; Liu, Y.; Wu, H.; Zheng, S.; Bao, S.; Ling, H.-Q. SKB1/PRMT5-Mediated Histone H4R3 Dimethylation of Ib Subgroup BHLH Genes Negatively Regulates Iron Homeostasis in Arabidopsis Thaliana. *Plant J.* **2014**, *77*, 209–221, doi:10.1111/tpj.12380.

45. Cheng, K.; Xu, Y.; Yang, C.; Ouellette, L.; Niu, L.; Zhou, X.; Chu, L.; Zhuang, F.; Liu, J.; Wu, H.; et al. Histone Tales: Lysine Methylation, a Protagonist in Arabidopsis Development. *J. Exp. Bot.* **2020**, *71*, 793–807, doi:10.1093/jxb/erz435.

46. Jiang, D.; Yang, W.; He, Y.; Amasino, R.M. Arabidopsis Relatives of the Human Lysine-Specific Demethylase1 Repress the Expression of FWA and FLOWERING LOCUS C and Thus Promote the Floral Transition. *Plant Cell* **2007**, *19*, 2975–2987, doi:10.1105/tpc.107.052373.

47. Ishihara, H.; Sugimoto, K.; Tarr, P.T.; Temman, H.; Kadokura, S.; Inui, Y.; Sakamoto, T.; Sasaki, T.; Aida, M.; Suzuki, T.; et al. Primed Histone Demethylation Regulates Shoot Regenerative Competency. *Nat. Commun.* **2019**, *10*, 1–15, doi:10.1038/s41467-019-09386-5.

48. Lu, F.; Cui, X.; Zhang, S.; Jenuwein, T.; Cao, X. Arabidopsis REF6 Is a Histone H3 Lysine 27 Demethylase. *Nat. Genet.* **2011**, *43*, 715–719, doi:10.1038/ng.854.

49. Yan, W.; Chen, D.; Smaczniak, C.; Engelhorn, J.; Liu, H.; Yang, W.; Graf, A.; Carles, C.C.; Zhou, D.-X.; Kaufmann, K. Dynamic and Spatial Restriction of Polycomb Activity by Plant Histone Demethylases. *Nat. Plants* **2018**, *4*, 681–689, doi:10.1038/s41477-018-0219-5.

50. Crevillén, P.; Yang, H.; Cui, X.; Greeff, C.; Trick, M.; Qiu, Q.; Cao, X.; Dean, C. Epigenetic Reprogramming That Prevents Transgenerational Inheritance of the Vernalized State. *Nature* **2014**, *515*, 587–590, doi:10.1038/nature13722.

51. Lu, F.; Cui, X.; Zhang, S.; Liu, C.; Cao, X. JMJ14 Is an H3K4 Demethylase Regulating Flowering Time in Arabidopsis. *Cell Res.* **2010**, *20*, 387–390, doi:10.1038/cr.2010.27.

52. Yang, H.; Han, Z.; Cao, Y.; Fan, D.; Li, H.; Mo, H.; Feng, Y.; Liu, L.; Wang, Z.; Yue, Y.; et al. A Companion Cell–Dominant and Developmentally Regulated H3K4 Demethylase Controls Flowering Time in Arabidopsis via the Repression of FLC Expression. *PLOS Genet.* **2012**, *8*, e1002664, doi:10.1371/journal.pgen.1002664.

53. Liu, P.; Zhang, S.; Zhou, B.; Luo, X.; Zhou, X.F.; Cai, B.; Jin, Y.H.; Niu, D.; Lin, J.; Cao, X.; et al. The Histone H3K4 Demethylase JMJ16 Represses Leaf Senescence in Arabidopsis. *Plant Cell* **2019**, *31*, 430–443, doi:10.1105/tpc.18.00693.

54. Huang, S.; Zhang, A.; Jin, J.B.; Zhao, B.; Wang, T.-J.; Wu, Y.; Wang, S.; Liu, Y.; Wang, J.; Guo, P.; et al. Arabidopsis Histone H3K4 Demethylase JMJ17 Functions in Dehydration Stress Response. *New Phytol.* **2019**, *223*, 1372–1387, doi:10.1111/nph.15874.

55. Saze, H.; Shiraishi, A.; Miura, A.; Kakutani, T. Control of Genic DNA Methylation by a JmjC Domain-Containing Protein in Arabidopsis Thaliana. *Science* **2008**, *319*, 462–465, doi:10.1126/science.1150987.

56. Fleury, D.; Himanen, K.; Cnops, G.; Nelissen, H.; Boccardi, T.M.; Maere, S.; Beemster, G.T.S.; Neyt, P.; Anami, S.; Robles, P.; et al. The Arabidopsis Thaliana Homolog of Yeast BRE1 Has a Function in Cell Cycle Regulation during Early Leaf and Root Growth. *Plant Cell* **2007**, *19*, 417–432, doi:10.1105/tpc.106.041319.

57. Dhawan, R.; Luo, H.; Foerster, A.M.; AbuQamar, S.; Du, H.-N.; Briggs, S.D.; Scheid, O.M.; Mengiste, T. HISTONE MONOUBIQUITINATION1 Interacts with a Subunit of the Mediator Complex and Regulates Defense against Necrotrophic Fungal Pathogens in Arabidopsis. *Plant Cell* **2009**, *21*, 1000–1019, doi:10.1105/tpc.108.062364.

58. Ma, S.; Tang, N.; Li, X.; Xie, Y.; Xiang, D.; Fu, J.; Shen, J.; Yang, J.; Tu, H.; Li, X.; et al. Reversible Histone H2B Monoubiquitination Fine-Tunes Abscisic Acid Signaling and Drought Response in Rice. *Mol. Plant* **2019**, *12*, 263–277, doi:10.1016/j.molp.2018.12.005.

59. Nassrallah, A.; Rougée, M.; Bourbousse, C.; Drevensek, S.; Fonseca, S.; Iniesto, E.; Ait-Mohamed, O.; Deton-Cabanillas, A.-F.; Zabulon, G.; Ahmed, I.; et al. DET1-Mediated Degradation of a SAGA-like Deubiquitination Module Controls H2Bub Homeostasis. *eLife* **2018**, *7*, e37892, doi:10.7554/eLife.37892.

60. Sridhar, V.V.; Kapoor, A.; Zhang, K.; Zhu, J.; Zhou, T.; Hasegawa, P.M.; Bressan, R.A.; Zhu, J.-K. Control of DNA Methylation and Heterochromatic Silencing by Histone H2B Deubiquitination. *Nature* **2007**, *447*, 735–738, doi:10.1038/nature05864.

61. Krichevsky, A.; Zaltsman, A.; Lacroix, B.; Citovsky, V. Involvement of KDM1C Histone Demethylase–OTLD1 Otubain-like Histone Deubiquitinase Complexes in Plant Gene Repression. *Proc. Natl. Acad. Sci.* **2011**, *108*, 11157–11162, doi:10.1073/pnas.1014030108.

62. Clapier, C.R.; Cairns, B.R. The Biology of Chromatin Remodeling Complexes. *Annu. Rev. Biochem.* **2009**, *78*, 273–304, doi:10.1146/annurev.biochem.77.062706.153223.

63. Han, S.-K.; Wu, M.-F.; Cui, S.; Wagner, D. Roles and Activities of Chromatin Remodeling ATPases in Plants. *Plant J.* **2015**, *83*, 62–77, doi:10.1111/tpj.12877.

64. Bezhani, S.; Winter, C.; Hershman, S.; Wagner, J.D.; Kennedy, J.F.; Kwon, C.S.; Pfluger, J.; Su, Y.; Wagner, D. Unique, Shared, and Redundant Roles for the *Arabidopsis* SWI/SNF Chromatin Remodeling ATPases BRAHMA and SPLAYED. *Plant Cell* **2007**, *19*, 403–416, doi:10.1105/tpc.106.048272.

65. Tang, X.; Hou, A.; Babu, M.; Nguyen, V.; Hurtado, L.; Lu, Q.; Reyes, J.C.; Wang, A.; Keller, W.A.; Harada, J.J.; et al. The Arabidopsis BRAHMA Chromatin-Remodeling ATPase Is Involved in Repression of Seed Maturation Genes in Leaves. *Plant Physiol.* **2008**, *147*, 1143–1157, doi:10.1104/pp.108.121996.

66. Yang, S.; Li, C.; Zhao, L.; Gao, S.; Lu, J.; Zhao, M.; Chen, C.-Y.; Liu, X.; Luo, M.; Cui, Y.; et al. The Arabidopsis SWI2/SNF2 Chromatin Remodeling ATPase BRAHMA Targets Directly to PINs and Is Required for Root Stem Cell Niche Maintenance. *Plant Cell* **2015**, *27*, 1670–1680, doi:10.1105/tpc.15.00091.

67. Wagner, D.; Meyerowitz, E.M. SPLAYED, a Novel SWI/SNF ATPase Homolog, Controls Reproductive Development in Arabidopsis. *Curr. Biol.* **2002**, *12*, 85–94, doi:10.1016/S0960-9822(01)00651-0.

68. Walley, J.W.; Rowe, H.C.; Xiao, Y.; Chehab, E.W.; Kliebenstein, D.J.; Wagner, D.; Dehesh, K. The Chromatin Remodeler SPLAYED Regulates Specific Stress Signaling Pathways. *PLOS Pathog.* **2008**, *4*, e1000237, doi:10.1371/journal.ppat.1000237.

69. Wu, M.-F.; Sang, Y.; Bezhani, S.; Yamaguchi, N.; Han, S.-K.; Li, Z.; Su, Y.; Slewinski, T.L.; Wagner, D. SWI2/SNF2 Chromatin Remodeling ATPases Overcome Polycomb Repression and Control Floral Organ Identity with the LEAFY and SEPALLATA3 Transcription Factors. *Proc. Natl. Acad. Sci.* **2012**, *109*, 3576–3581, doi:10.1073/pnas.1113409109.

70. Mlynárová, L.; Nap, J.-P.; Bisseling, T. The SWI/SNF Chromatin-Remodeling Gene AtCHR12 Mediates Temporary Growth Arrest in Arabidopsis Thaliana upon Perceiving Environmental Stress. *Plant J.* **2007**, *51*, 874–885, doi:10.1111/j.1365-313X.2007.03185.x.

71. Sang, Y.; Silva‐Ortega, C.O.; Wu, S.; Yamaguchi, N.; Wu, M.-F.; Pfluger, J.; Gillmor, C.S.; Gallagher, K.L.; Wagner, D. Mutations in Two Non-Canonical Arabidopsis SWI2/SNF2 Chromatin Remodeling ATPases Cause Embryogenesis and Stem Cell Maintenance Defects. *Plant J.* **2012**, *72*, 1000–1014, doi:10.1111/tpj.12009.

72. Li, G.; Liu, S.; Wang, J.; He, J.; Huang, H.; Zhang, Y.; Xu, L. ISWI Proteins Participate in the Genome-Wide Nucleosome Distribution in Arabidopsis. *Plant J.* **2014**, *78*, 706–714, doi:10.1111/tpj.12499.

73. Luo, Y.-X.; Hou, X.-M.; Zhang, C.-J.; Tan, L.-M.; Shao, C.-R.; Lin, R.-N.; Su, Y.-N.; Cai, X.-W.; Li, L.; Chen, S.; et al. A Plant-Specific SWR1 Chromatin-Remodeling Complex Couples Histone H2A.Z Deposition with Nucleosome Sliding. *EMBO J.* **2020**, *n/a*, e102008, doi:10.15252/embj.2019102008.

74. Shen, Y.; Devic, M.; Lepiniec, L.; Zhou, D.-X. Chromodomain, Helicase and DNA-Binding CHD1 Protein, CHR5, Are Involved in Establishing Active Chromatin State of Seed Maturation Genes. *Plant Biotechnol. J.* **2015**, *13*, 811–820, doi:10.1111/pbi.12315.

75. Zou, B.; Sun, Q.; Zhang, W.; Ding, Y.; Yang, D.-L.; Shi, Z.; Hua, J. The Arabidopsis Chromatin-Remodeling Factor CHR5 Regulates Plant Immune Responses and Nucleosome Occupancy. *Plant Cell Physiol.* **2017**, *58*, 2202–2216, doi:10.1093/pcp/pcx155.

76. Carter, B.; Bishop, B.; Ho, K.K.; Huang, R.; Jia, W.; Zhang, H.; Pascuzzi, P.E.; Deal, R.B.; Ogas, J. The Chromatin Remodelers PKL and PIE1 Act in an Epigenetic Pathway That Determines H3K27me3 Homeostasis in Arabidopsis. *Plant Cell* **2018**, *30*, 1337–1352, doi:10.1105/tpc.17.00867.

77. Jing, Y.; Zhang, D.; Wang, X.; Tang, W.; Wang, W.; Huai, J.; Xu, G.; Chen, D.; Li, Y.; Lin, R. Arabidopsis Chromatin Remodeling Factor PICKLE Interacts with Transcription Factor HY5 to Regulate Hypocotyl Cell Elongation. *Plant Cell* **2013**, *25*, 242–256, doi:10.1105/tpc.112.105742.

78. Zhang, H.; Rider, S.D.; Henderson, J.T.; Fountain, M.; Chuang, K.; Kandachar, V.; Simons, A.; Edenberg, H.J.; Romero-Severson, J.; Muir, W.M.; et al. The CHD3 Remodeler PICKLE Promotes Trimethylation of Histone H3 Lysine 27. *J. Biol. Chem.* **2008**, *283*, 22637–22648, doi:10.1074/jbc.M802129200.

79. Zhang, D.; Jing, Y.; Jiang, Z.; Lin, R. The Chromatin-Remodeling Factor PICKLE Integrates Brassinosteroid and Gibberellin Signaling during Skotomorphogenic Growth in Arabidopsis. *Plant Cell* **2014**, *26*, 2472–2485, doi:10.1105/tpc.113.121848.

80. Perruc, E.; Kinoshita, N.; Lopez‐Molina, L. The Role of Chromatin-Remodeling Factor PKL in Balancing Osmotic Stress Responses during Arabidopsis Seed Germination. *Plant J.* **2007**, *52*, 927–936, doi:10.1111/j.1365-313X.2007.03288.x.

81. Yang, R.; Hong, Y.; Ren, Z.; Tang, K.; Zhang, H.; Zhu, J.-K.; Zhao, C. A Role for PICKLE in the Regulation of Cold and Salt Stress Tolerance in Arabidopsis. *Front. Plant Sci.* **2019**, *10*, doi:10.3389/fpls.2019.00900.

82. Deal, R.B.; Topp, C.N.; McKinney, E.C.; Meagher, R.B. Repression of Flowering in Arabidopsis Requires Activation of FLOWERING LOCUS C Expression by the Histone Variant H2A.Z. *Plant Cell* **2007**, *19*, 74–83, doi:10.1105/tpc.106.048447.

83. Zhang, C.; Cao, L.; Rong, L.; An, Z.; Zhou, W.; Ma, J.; Shen, W.-H.; Zhu, Y.; Dong, A. The Chromatin-Remodeling Factor AtINO80 Plays Crucial Roles in Genome Stability Maintenance and in Plant Development. *Plant J.* **2015**, *82*, 655–668, doi:10.1111/tpj.12840.

84. Heo, J.B.; Sung, S. Vernalization-Mediated Epigenetic Silencing by a Long Intronic Noncoding RNA. *Science* **2011**, *331*, 76–79, doi:10.1126/science.1197349.
